# High-throughput sequencing analysis of community diversity and functional structure of endophytic bacteria in edible vegetable crops: potential implication on plant microbiological quality

**DOI:** 10.1007/s13205-025-04380-9

**Published:** 2025-06-17

**Authors:** Adekunle Raimi, Rasheed Adeleke

**Affiliations:** Unit for Environmental Sciences and Management, North-West University, Potchefstroom, 2520 South Africa

**Keywords:** Microbiome, Bacterial endophytes, Agroecosystem, Fertilizer, Vegetables, Enzyme

## Abstract

This study evaluated the diversity and functional structure of endophytic bacterial communities residing within four common leafy vegetables: *Brassica oleracea, Lactuca sativa, Allium cepa,* and *Spinacia oleracea*, cultivated under organic (OF) and conventional (CF) farming systems. Utilizing high-throughput 16S rRNA gene sequencing and the PICRUSt2 pipeline, the research assessed the influence of plant species, organ (leaf/root), and fertilizer type on these microbial communities. Findings revealed that plant species and organ type significantly shaped endophytic bacterial community composition and diversity. Onion communities were distinct, and roots exhibited higher diversity and richness compared to leaves. Fertilizer type significantly impacted overall bacterial diversity, with CF farms showing higher diversity than OF. Microbial network analysis identified keystone taxa, including network hubs like *Serratia* and *Streptomyces*, and module hubs like *Solirubrobacter*, *Corynebacterium*, and *Mycobacterium*. Functional predictions indicated diverse metabolic capabilities, with organ type significantly affecting pathway abundance (leaves enriched in carbohydrate degradation, roots in nutrient metabolism/degradation). OF farms showed higher predicted abundance of some potential virulence pathways, while CF farms had higher abundance of certain biotechnological pathways. Vegetable nutrient content significantly correlated with both bacterial community composition and predicted metabolic pathways. This study highlights the complex interplay between farming practices, plant factors, endophytic microbiomes, and their functional potential, underscoring implications for vegetable microbiological quality and potential human health.

**Supplementary Information:**

The online version contains supplementary material available at 10.1007/s13205-025-04380-9.

## Introduction

Leafy vegetables are essential components of a healthy human diet, supplying vital nutrients such as dietary fiber, folate, potassium, and various vitamins (Kumar et al. 2020; Mungofa et al. 2022). Beyond their intrinsic nutritional values, these vegetables host endophytic microbes within their tissues, which play essential roles in promoting host plant health (Nithya and Babu 2017; Chaudhary et al. 2022). Despite their widespread cultivation and consumption, vegetables are less explored for their bacterial endophytic communities and their influence on plant nutritional values.

Endophytic bacteria have various agroecological roles contributing to sustainable agriculture and ecosystem functioning (Adeleke et al. 2019; Santos and Olivares 2021; Rani et al. 2022). In addition, they produce numerous biochemical compounds and enzymes potentially important for human health (Singh et al. 2017; Raimi and Adeleke 2021). Gaining insight into the community composition and functional diversity of these endophytes can provide crucial understanding of plant–microbe interactions and their potential for biotechnological applications.

The increasing global demand for vegetables has led to intensified agricultural practices (Mason-D’Croz et al. 2019). A primary strategy for enhancing soil nutrient richness and supporting increased vegetable production involves the application of organic and conventional fertilizers (Ngetich et al. 2012; Montgomery and Biklé 2021). Organic fertilizers, derived from sources such as green waste, animal manure, and compost, offer sustainable approaches for improving plant health and quality (Bhunia et al. 2021). Similarly, conventional farming employs chemical fertilizers, pesticides, and fungicides to boost plant productivity. However, both types of fertilizers can have detrimental environmental impacts, including soil structure degradation, loss of microbial communities, and pollution of air and water bodies. They shape the diversity and functional structure of associated soil and plant microbiomes, influencing their metabolism, enzyme activities, and genetic repertoire (Pittarello et al. 2021; Zhang et al. 2022). Furthermore, farm practices are associated with the prevalence of antibiotic-resistant genes (ARGs) in soil, microbes, and plants (Flandroy et al. 2018; Ager et al. 2023). This connection poses potential implications for human nutrition via the microbiological quality of plant food, especially vegetables frequently consumed raw (Wicaksono et al. 2023). Under the One-Health concept, these practices may potentially affect human nutrition through the microbiological quality of plant food, especially vegetables which are eaten raw (Wang et al. 2015; Scaccia et al. 2021; Chen et al. 2023). Therefore, it is crucial to comprehensively evaluate the impact of organic and conventional fertiliser (CF) application on endophytic bacterial community composition and functions as well as their potential effects on plant nutritional quality.

Endophytic bacteria contribute significantly to plant growth and development, enhancing processes such as nutrient uptake, resistance to pests and pathogens, and tolerance to biotic and abiotic stress (Afzal et al. 2019; Nanda et al. 2019; Chaudhary et al. 2022). Specific bacterial endophytes, including *Acinetobacter*, *Serratia*, *Bacillus*, and *Stenotrophomonas*, are known to produce indole acetic acid (IAA) and solubilize phosphate, exhibiting antagonistic potential against pathogens like *Colletotrichum* and *Fusarium* (Wang et al. 2016; Raimi and Adeleke 2023). A better knowledge of the mechanisms that drive these benefits under different ecosystems is crucial to microbial technology for improving crop yield and health. Factors, including geographical locations, plant species and organs (Christian et al. 2016) and agronomic practices, such as fertilizer types, irrigation and soil tillage impact soil and plant ecosystems (Azarbad 2022), which may consequently shape the bacterial endophyte diversity and functional structure (Xia et al. 2015). However, these ecological and biologic factors have received less attention (Wagner et al. 2016), especially as it relates to vegetable production.

Characterization of microbial community structures traditionally relies on compositional and diversity metrics (An et al. 2019; Hoch et al. 2019). However, recent advancements in understanding the dynamics of inter-taxa interactions within a community, often using network analysis, offer further insights into microbial community structure and functions (Pan et al. 2021). Taxa that exhibit strong interactions and exert significant influence on microbial structure and function, regardless of their abundance, are termed keystone taxa. Keystone taxa exist in various ecosystems and perform diverse ecological functions (Banerjee et al. 2018a, b; Yang et al. 2021).Advancing our understanding of microbial interactions can reveal critical mechanisms driving plant–microbe associations and pinpoint keystone species with potential impacts on plant endophytic communities and ultimately human nutrition. This study employed high-throughput 16S rRNA gene sequencing to compare the abundance and diversity of bacterial endophytes in vegetable crops across different plant species and fertiliser types (organic and conventional). By using network analysis, keystone taxa with unique functions that potentially impact plant nutritional quality are identified. Furthermore, the study examined the correlation between bacterial community diversity and functional profiles across plant species and fertilizer types. The functional profile was predicted using the Phylogenetic Investigation of Communities by reconstruction of unobserved States 2 (PICRUSt2) pipeline (version 2.3.0) (Douglas et al. 2020). Based on this framework, we hypothesized that, a distinctive community structure exists that varies across vegetable species, organs, and fertiliser types, indicating that microbial communities and functions exhibit patterns influenced by ecological factors. Understanding such patterns in plant–microbe interactions is crucial for obtaining valuable information about agronomic strategies that best support sustainable agriculture and could improve plant health and human nutritional management. In addition, we also worked on a secondary hypothesis based on the data analysis that only focused on potential functions of the endophytes. This hypothesis states that endophytic bacterial metabolism pathways are significantly impacted by plant species, nutrient content, and fertilizer types.

## Materials and methods

### Study site and vegetable crop sampling

**Fig. 1:**
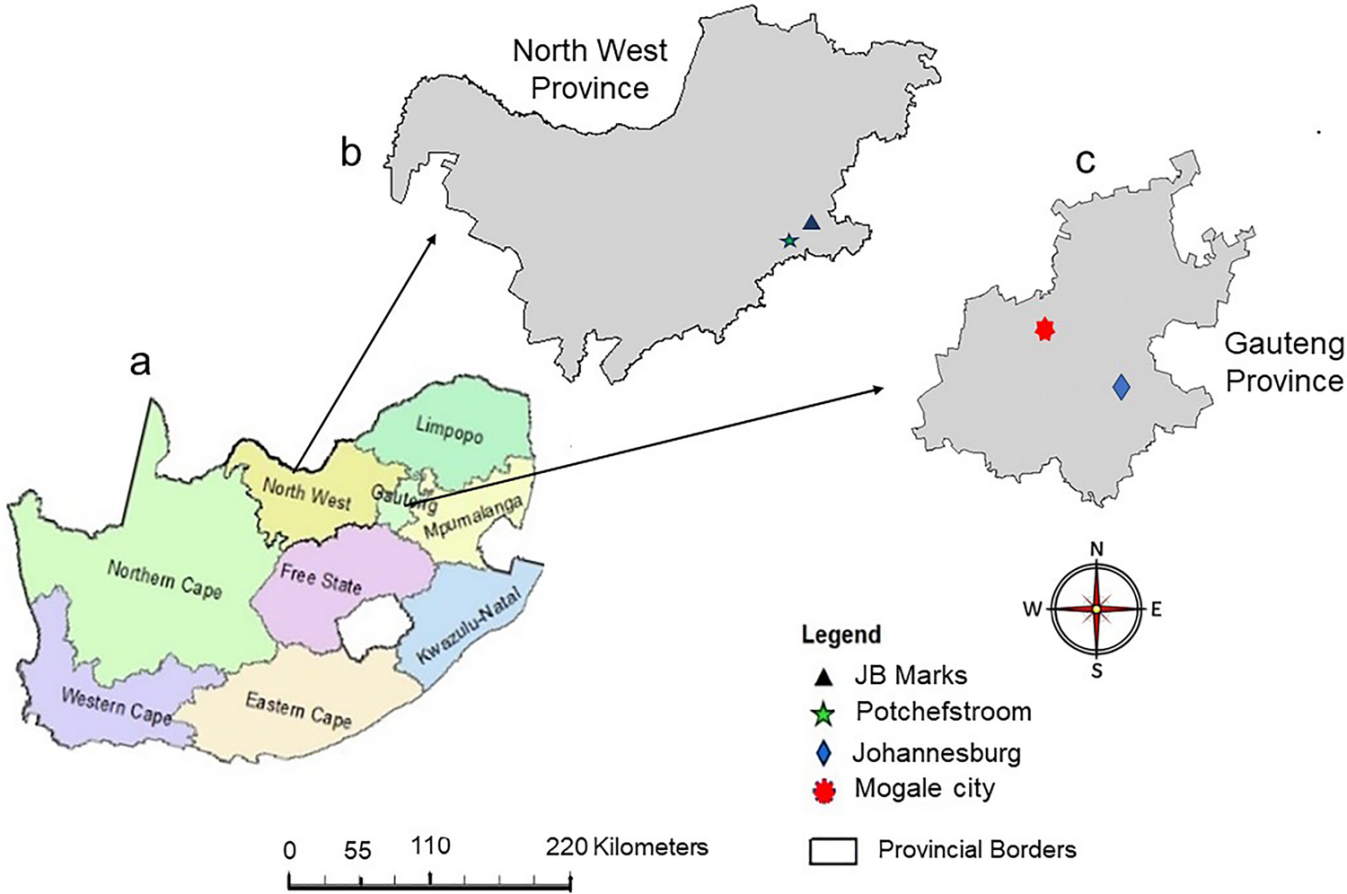
Location of sampling sites. This figure displays the geographical locations of the four vegetable-production farms sampled for this study. Map **a** shows South Africa, map **b** highlights the North West province, and map **c** shows the Gauteng province. Five vegetable samples were collected from two distinct farms in each province. The North West province farms, Small Farm (SF) and Big Farm (BF), are situated in Potchefstroom and JB Marks municipalities, respectively, and are categorized as OF farms utilizing plant waste and animal manure. The Gauteng province farms, Johannesburg Farm (JF) and Third Farm (TF), are located in Bertrams and Mogale City, respectively, and are classified as conventional fertiliser (CF) farms employing chemical fertilizers and pesticides. From Johannesburg (dark blue colored prism shape) and Mogale (red colored hexagon shape) cities

Four vegetable-production farms situated in the North West and Gauteng provinces of South Africa were used for this study. The North West farms, SF (26° 47′ 43.5″ S 27° 02′ 18.3″ E) and BF (26° 42′ 55.0″ S 27° 04′ 59.6″ E) are located in Potchefstroom, JB Marks municipality, while the Gauteng province farms, JF (26° 11′ 40.1″ S 28° 03′ 58.3″ E) and TF (26° 03′ 16.0″ S 27° 40′ 13.3″ E) are situated in Bertrams and Mogale city, respectively (Fig. 1). The geographic location and climate parameters are presented in Supplementary Table 1. Soil classification for the North West and Gauteng province farms is clayey and sandy loam, respectively. To improve soil nutrient richness and plant productivity, the North West farms use plant waste, cow, goat, and poultry manure and are categorized as organic fertiliser (OF) farms, while the Gauteng farms use chemical fertilizers and pesticides, and are classified as conventional fertiliser (CF) farms in the present study.

Vegetable crops, cabbage (*Brassica oleracea*)*,* lettuce (*Lactuca sativa*)*,* Onion (*Allium cepa*) and spinach (*Spinacia oleracea*) were sampled from the farms. To ensure unbiased sampling, healthy plants of the same age and size were sampled based on visual inspection. Using a sterile garden fork, a whole vegetable crop was carefully uprooted without damaging the tissues and shaken vigorously to remove bulk soil from the roots. From each farm, five biologic replicates for each vegetable were randomly sampled and immediately placed in sterile zip-lock bags and transported to the laboratory on ice to maintain microbiological integrity. The samples were washed with distilled water and the phyllosphere (leaf and stem) and root were carefully lacerated using a sterile blade. To eliminate the interference of viable and nonviable epiphytic microbes and obtain only endophytic bacteria, plant tissues were sterilised following the method described by (McKinnon 2016).

### Bacterial 16S rRNA gene library preparation

Total genomic DNA was extracted from the plant organs (pulverised phyllosphere and root) using Qiagen DNeasy Plant kits (QIAGEN^®^, Hilden, Germany), following the manufacturer’s instructions. The DNA was quantified with a Nanodrop™−1000 (Nanodrop Technologies lnc., Wilmington, USA), while the integrity and the size were checked in a 1% agarose gel electrophoresis. The 16S rRNA gene was amplified in a polymerase chain reaction using Illumina barcoded primers targeting the V3-V4 region (Table 1). The library preparation was performed as described by (van Wyk et al. 2017). Paired end (250 bp) sequencing of the libraries was performed on Illumina MiSeq sequencer using Nextera v3 kit (Illumina Inc., San Diego, USA) at the Sequencing facility of the Unit for Environmental Sciences and Management, North-West University, Potchefstroom, South Africa.

**Table 1:** Illumina primers

Primer name	16S variable region	Primer sequence 5′–3′	Amplicon size	References
341 Forward	V3-V4	CCTACGGGNGGCWGCAG	464	Klindworth et al. (2013)
805 Reverse	V3-V4	GACTACHVGGGTATCTAATCC		

### Bioinformatics analysis

After demultiplexing and trimming of barcodes using MiSeq Reporter software (Illumina Inc., San Diego, USA), the quality of the reads was checked with FastQC v. 0.11.9 (Babraham Bioinformatics, UK), and trimmed using Trimmomatic software v. 0.38 (Bolger et al. 2014). The DADA2 plugin in QIIME2 was then utilized to correct for errors, remove chimeras and cluster the sequences at 100% similarities to amplicon sequence variants (ASVs) as described in the supplementary information (Supplementary Text S1). Taxonomic classification, phylogenetic relationship, and alpha and beta diversity analyses were performed in QIIME2 and R software v. 4.1.3 (R Development Core Team 2018). The ASV countable obtained were rarefied to an even depth of 7340 in R software. Alpha diversity, based on Observed ASVs, Chao1, Shannon, Simpson and Pielou’s evenness were used for community diversity, richness, and uniformity analyses. Sequencing depth adequacy in each sample was evaluated with the Good’s coverage index and rarefaction curve analysis. Endophytic bacterial community abundance and composition were calculated and compared between the fixed factors (plant species, organ, and fertiliser types) using beta diversity, based on weighted and unweighted unifrac distances in R studio. Using the ggVennDiagram package in R, a Venn diagram was made to show the presence and distribution of unique and shared species. Core species are the ‘stable’ part of the community exhibiting key functions that impact host physiologic performance and may be associated with the unique or shared species (Alibrandi et al. 2020).

### Prediction of endophytic bacterial functional profile

The functional metabolic profile of the endophytes was computed with the Phylogenetic investigation of communities by reconstruction of unobserved states 2 (PICRUSt2) pipeline (version 2.3.0) (Douglas et al. 2020). The pipeline was executed in QIIME2 software using the ASV sequence and abundance table generated against the SILVA rRNA (138) database as an input file. The output files included gene family and pathway abundances (Douglas et al. 2020). The average Nearest Sequenced Taxon Index (NSTI) score was 0.134 ± 0.024 (± SD) and 3% of ASVs having NSTI above the maximum cut-off score of 2 were expunged. The non-metric multidimensional scaling (NMDS) and the redundancy analysis (RDA) plot were used to investigate the effects of plant species, plant nutrient content and fertilizer type on the endophytic bacterial community functions. A subset of essential pathways involved in key ecosystem functions contributing to metabolism, degradation, biosynthesis, and decomposition and relating to plant nutritional quality were analyzed.

### Microbial co-occurrence network analysis

Microbial network analysis was performed to examine the interactions among bacterial communities across the fixed factors, using the random matrix theory (RMT) in the molecular ecological network analysis (MENA) pipeline (http://ieg2.ou.edu/MENA/). Detailed procedures for constructing and analyzing networks are well elucidated in previous studies (Zhou et al. 2011; Deng et al. 2012); however, major steps are summarized in the supplementary information (Supplementary Text S2). Unnormalized data were used for the analysis and ASVs present in at least 20% of the samples were considered to ensure statistical consistency and reliability. Microbial community interactions were clustered into modules based on their within (Zi) and among (closeness) (Pi) module connectivity criteria, causing nodes to be grouped into four sub-categories: peripherals, connectors, module and network hubs, using the threshold values for Zi and Pi as follows: peripherals (Zi < 2.5; Pi < 0.62), connectors (Zi < 2.5; Pi > 0.62), network hubs (Zi > 2.5; Pi > 0.62) and module hubs (Zi > 2.5; Pi < 0.62) (Guimera and Amaral 2005). Constructed networks were visualized using the Gephi v 0.9.2 interactive tool. The network analysis details are available in Supplementary Text S3.

### Statistical analyses

R software (version 4.1.3) (R Development Core Team 2018) was used for the statistical analyses, with the significant level set at probability, *P* < 0.05. Data normality was verified using the Shapiro–Wilk test and non-normally distributed data were transformed using log, square root, or sine function. Normally and non-normally distributed data were analyzed using parametric and non-parametric tests, respectively. Mantel test was used to assess the correlations between vegetable nutrient content, bacterial community composition and metabolic pathways. The relative proportion of each ASV count was log-transformed (log (*x*) + 1, where *x* > 0) with the “decostand ()” function in the vegan package (version 2.6–4) of R for multivariate analyses. Bacterial community structure in multivariate space was visualized with a nonmetric multidimensional scaling (NMDS) using the vegan and dendextend (v. 1.12.0) packages in R. Differences between groups were tested with weighted and unweighted unifrac distances dissimilarity using permutational multivariate analysis of variance (PERMANOVA) and Permutational test for homogeneity of multivariate dispersions (PERMDISP). The linear discriminant analysis (LDA) effect size (LEfSe) was used to detect the most differentially abundant community and pathways (Kruskal–Wallis test, *P* < 0.05, LDA score > 2) between sample groups. Differentially abundant bacterial communities that are statistically significant were visualized as bar plots using the microeco package (Liu et al. 2021). An indicator species analysis was performed for the predicted pathways to detect the most discriminatory pathways between vegetable species and fertiliser types. To determine the plant nutrient content that best explains the variations in the microbial community composition and pathways across the sample group, RDA plot was made using an automatic stepwise model selection (“ordistep ()”) in the vegan package of R. Variables with multicollinearity (variance inflation factor > 10) were excluded from the final plot. Log-transformed nutrient data and bacterial species composition relative counts (> 1% genus taxa) were used for the RDA plot.

### Data availability

The 16S rRNA gene sequence data is available in the sequence read archives (SRA) of the National Centre for Biotechnological Information as part of a BioProject under the SRA accession number PRJNA1037753 (https://www.ncbi.nlm.nih.gov/bioproject/1037753).

## Results

### Comparison of the nutrient content of vegetable crops cultivated under organic fertilizer and conventional systems

The vegetable nutrient content varies between plant species and fertilizer types (Table 2). Compared to other vegetables, spinach had the highest amount of nutrients, including N, Mg, Na, Cu, and Mn across all the farms and fertiliser types. Similarly, spinach had the highest value for Fe, and Zn in all the farms except in farms SF and TF where lettuce and onion had the highest mean values, respectively. For Ca, cabbage was highest in both OF and CF farm vegetables with a value of 2.14 and 3.18 mg/100 g, respectively (Table 2). Onion had the highest amount of P and K in organic farms, while spinach and lettuce had the highest, respectively in CF farms. The %S was not very high across the farms, with the least amount found in lettuce, while the highest (0.53%) was in cabbage, except for spinach in farm SF. The nutrient content varied significantly (*P* < 0.05) between vegetable species for each farm. The main effect of plant species was significant for the nutrient elements except for P (Supplementary Fig. 1). Similarly, regardless of the plant species, the nutrients were significantly (Mann *U* Whitney test, *P* < 0.05) different across OF and CF farms, except for B, N, Na, Mg and S. The main effect of organ types was statistically not significant (*P* > 0.05). There was a significant (ANOVA *P* < 0.05) interaction effect between plant species and fertilizer (organic or conventional) type except for Zn, while the interactions between plant species and organ type were found not to be significant (*P* > 0.05). Due to the unbalanced sample size across the plant species, the composite effects of the interactions between farms, plant species, and fertilizer type were not considered.

**Table 2:** Nutrient content of vegetable crops from different farm locations

	Farm	Plant	(mg/100 g)											
			N	P	K	Ca	Mg	Na	Cu	Fe (mg/kg)	Zn	Mn	B	S (%)
Organic farm	BF	Cabbage	3.16 ± 0.15^b^	0.46 ± 0.02^b^	3.41 ± 0.03^b^	2.14 ± 0.04^a^	0.75 ± 0.03^b^	0.47 ± 0.01^c^	8.17 ± 0.27^b^	50.15 ± 0.56^c^	24.05 ± 0.58^c^	36.23 ± 0.26^b^	19.29 ± 0.28^c^	0.48 ± 0.01^a^
	BF	Onion	3.34 ± 0.05^b^	0.76 ± 0.01^a^	4.07 ± 0.02^a^	1.11 ± 0.04^b^	0.37 ± 0.02^c^	2.36 ± 0.03^b^	6.98 ± 0.09^c^	107.65 ± 0.57^b^	30.82 ± 0.28^b^	11.18 ± 0.11^c^	21.58 ± 0.41^b^	0.27 ± 0.02^b^
	BF	Spinach	4.31 ± 0.07^a^	0.33 ± 0.02^c^	3.33 ± 0.07^b^	0.69 ± 0.05^c^	1.10 ± 0.01^a^	3.26 ± 0.06^a^	28.53 ± 0.51^a^	140.56 ± 0.52^a^	62.54 ± 0.51^a^	49.04 ± 0.67^a^	28.51 ± 0.49^a^	0.27 ± 0.02^b^
	SF	Cabbage	2.14 ± 0.02^c^	0.27 ± 0.02^b^	2.73 ± 0.05^c^	0.50 ± 0.01^c^	0.21 ± 0.01^b^	0.35 ± 0.01^d^	3.55 ± 0.05^d^	87.32 ± 0.37^c^	19.69 ± 0.3^c^	16.18 ± 0.28^c^	16.24 ± 0.47^b^	0.33 ± 0.02^b^
	SF	Lettuce	2.33 ± 0.08^b^	0.49 ± 0.01^a^	3.51 ± 0.03^b^	0.35 ± 0.03^d^	0.17 ± 0.01^c^	0.98 ± 0.02^b^	7.48 ± 0.08^b^	106.0 ± 1.00^a^	45.0 ± 1.00^b^	32.15 ± 0.26^b^	11.52 ± 0.08^c^	0.12 ± 0.03^d^
	SF	Onion	1.33 ± 0.03^d^	0.27 ± 0.02^b^	1.57 ± 0.02^d^	0.57 ± 0.02^b^	0.21 ± 0.01^b^	0.73 ± 0.01^c^	4.92 ± 0.39^c^	88.27 ± 0.21^b^	18.6 ± 0.37^d^	31.48 ± 0.51^b^	16.77 ± 0.26^b^	0.18 ± 0.01^c^
	SF	Spinach	4.26 ± 0.02^a^	0.47 ± 0.02^a^	3.79 ± 0.04^a^	0.93 ± 0.02^a^	0.93 ± 0.02^a^	2.96 ± 0.02^a^	13.43 ± 0.41^a^	82.00 ± 1.00^d^	66.99 ± 0.88^a^	99.00 ± 1.00^a^	31.17 ± 0.77^a^	0.37 ± 0.01^a^
Conventional farm	JF	Cabbage	3.62 ± 0.12^a^	0.59 ± 0.03^b^	4.21 ± 0.21^c^	3.18 ± 0.21^a^	0.74 ± 0.05^b^	0.34 ± 0.02^c^	3.64 ± 0.32^c^	59.83 ± 1.76^c^	51.38 ± 0.13^c^	16.43 ± 0.2^c^	17.64 ± 0.56^c^	0.53 ± 0.04^a^
	JF	Lettuce	2.84 ± 0.09^b^	0.63 ± 0.03^a^	6.49 ± 0.09^a^	1.72 ± 0.06^b^	0.62 ± 0.02^c^	1.09 ± 0.13^b^	9.53 ± 0.11^b^	108.95 ± 0.93^b^	85.64 ± 0.33^b^	35.85 ± 0.31^b^	25.65 ± 0.57^a^	0.18 ± 0.02^b^
	JF	Spinach	3.61 ± 0.11^a^	0.52 ± 0.04^b^	5.89 ± 0.04^b^	1.32 ± 0.05^c^	1.06 ± 0.04^a^	2.21 ± 0.03^a^	11.92 ± 0.35^a^	172.32 ± 2.11^a^	178.0 ± 0.55^a^	65.18 ± 0.76^a^	23.74 ± 0.28^b^	0.16 ± 0.01^b^
	TF	Cabbage	2.54 ± 0.11^b^	0.46 ± 0.04^b^	3.76 ± 0.21^c^	0.55 ± 0.04^d^	0.23 ± 0.02^c^	0.57 ± 0.04^c^	3.49 ± 0.04^c^	73.65 ± 0.31^c^	24.52 ± 0.5^c^	41.65 ± 0.31^c^	13.01 ± 0.05^d^	0.43 ± 0.06^a^
	TF	Lettuce	2.16 ± 0.11^c^	0.33 ± 0.02^c^	4.2 ± 0.05^b^	1.36 ± 0.04^a^	0.43 ± 0.02^b^	1.72 ± 0.03^b^	5.16 ± 0.30^b^	126.31 ± 1.43^b^	20.0 ± 1.00^d^	111.17 ± 1.76^b^	20.32 ± 0.59^b^	0.13 ± 0.05^c^
	TF	Onion	2.14 ± 0.07^c^	0.49 ± 0.03^b^	3.4 ± 0.12^d^	0.60 ± 0.01^c^	0.23 ± 0.02^c^	0.59 ± 0.02^c^	5.21 ± 0.29^b^	75.16 ± 1.04^c^	39.23 ± 0.44^a^	30.34 ± 0.82^d^	15.74 ± 0.71^c^	0.29 ± 0.01^b^
	TF	Spinach	3.47 ± 0.18^a^	0.85 ± 0.04^a^	5.43 ± 0.18^a^	0.88 ± 0.02^b^	0.66 ± 0.02^a^	2.32 ± 0.02^a^	10.62 ± 0.55^a^	190.33 ± 0.62^a^	33.52 ± 0.51^b^	151.65 ± 0.57^a^	26.77 ± 0.26^a^	0.27 ± 0.01^b^

Values are mean ± SD (*n* = 5). Except for sulfur (S), which is in % proportion, the nutrient content was measured in mg/100 mg. Fe was measured in mg/kg. Values with different superscript letters for each farm (across the vegetables for each column) are significantly different based on the non-parametric Kruskal–Wallis test or the parametric ANOVA test

*CEC* cation exchange capacity, *EC* electrical conductivity, *OM* organic matter, *TOC* total organic carbon

### Alpha diversity and endophytic bacterial structure in multivariate space

**Fig. 2:**
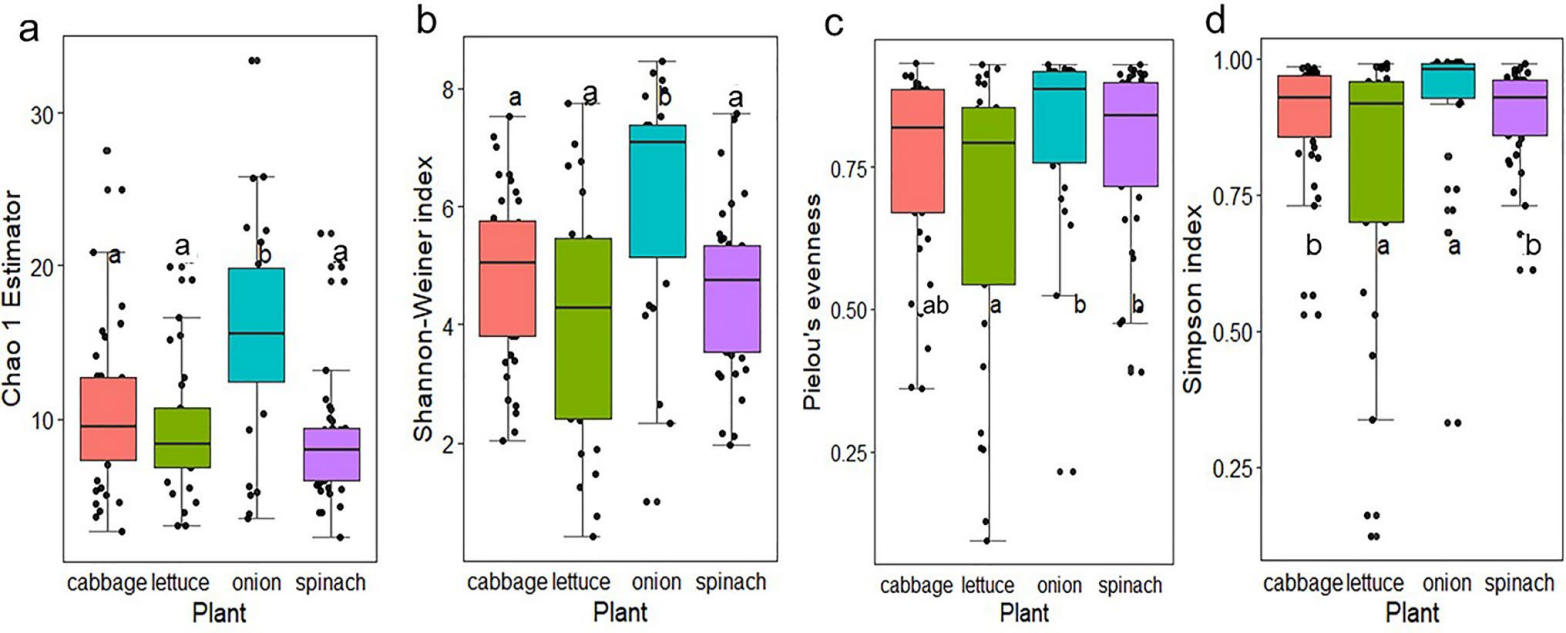
Alpha diversity of endophytic bacterial communities across vegetable species. This figure illustrates the alpha diversity metrics of endophytic bacterial communities observed in the four leafy vegetables studied: cabbage (*Brassica oleracea*), lettuce (*Lactuca sativa*), onion (*Allium cepa*), and spinach (*Spinacia oleracea*). Plots show **a** Chao1, (b) Shannon–Weiner (H′), **c** Pielou’s evenness, and **d** Simpson index. The results indicate that these alpha diversity measures significantly varied across vegetable species, plant organs, and fertiliser types (Kruskal–Wallis test, Mann–Whitney *U* test, *P* < 0.05). Error bars denote the standard deviation. Letters above the bars indicate significant differences (*P* < 0.05) based on the Tukey HSD comparison test, with the same letters indicating no significant difference. Onion demonstrated higher core abundance and richness compared to other plant species

The alpha diversity, including Chao1, Shannon–Weiner, Simpson and Pielou’s evenness significantly (Kruskal–Wallis test, Mann–Whitney *U* test, *P* < 0.05) vary across vegetable species (Fig. 2a–d), organ, and fertilizer type (Supplementary Fig. 2, Supplementary Table 2). Results reveal that the abundance and richness of bacterial endophytes were lower in the leaves compared to the roots. The phylogenetic diversity measure was not significantly different across the plant species (Supplementary Fig. 3a) and organ types (Supplementary Table 3). However, the fertiliser types showed a significant difference with a higher mean observed in CF farms. The core abundance was significantly different across the plant types (Supplementary Fig. 3b). Onions had a higher abundance and richness compared to other plant species. The interaction effects of all fixed factors did not influence the differences in alpha diversity, except for the Shannon-Weiner index, which was found significant [general linear model (glm), *P* = 0.0423] (Supplementary Table 4).

**Fig. 3:**
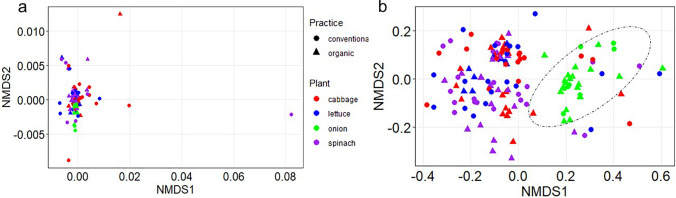
Endophytic bacterial community structure visualized by non-metric multidimensional scaling (NMDS). This figure presents the dissimilarity in endophytic bacterial communities across different vegetable species and fertilizer types using non-metric multidimensional scaling (NMDS) plots. The plots are based on **a** weighted Unifrac and **b** unweighted Unifrac distances. The NMDS ordination reveals a substantial overlap in the bacterial communities of cabbage, lettuce, and spinach, while the onion samples tend to cluster separately, indicating a distinct community structure for onion. Despite reduced homogeneity in sample dispersion for plant species, onion remained significantly different from other plants. PERMANOVA analysis showed that both plant species and organ types significantly influenced the bacterial community composition (*P* < 0.05). The 95% confidence ellipses in plot **b** represent the standard error in multivariate space for each group. Fertilizer type did not result in distinct clustering patterns in the NMDS plot

The NMDS ordination plot reveals a substantial overlap in the bacterial communities of cabbage, lettuce and spinach, while onion samples somewhat cluster separately (Fig. 3a, b). Despite reduced homogeneity of sample dispersion in the plant species group, onion remained significantly different from other plants. The PERMANOVA analysis suggests that plant species and organs had significant effects on the bacterial community composition (PERMANOVA; *P* < 0.05), with the dispersion test (PERMDISP, *P* > 0.05, Supplementary Table 5) that showed homogeneity for plant species, and thus, the bacterial community variations may be due to plant species type. However, the significant (PERMDISP, *P* < 0.05) dispersion for organ factor may have influenced the variations observed in the bacterial communities for this group. The only significant interaction effect was between plant species and organs for the unweighted unifrac (PERMANOVA R2 = 2.19%, *P* < 0.036; PERMDISP *P* = 0.454) (Fig. 3b). In addition, some fixed factor interactions only accounted for variations in the bacterial community (PERMDISP, *P* > 0.05) without significantly impacting the community composition (PERMANOVA, *P* > 0.05, Supplementary Table 5).

### Comparative analysis of dominant and differentially abundant phylotype across fixed factors

**Fig. 4:**
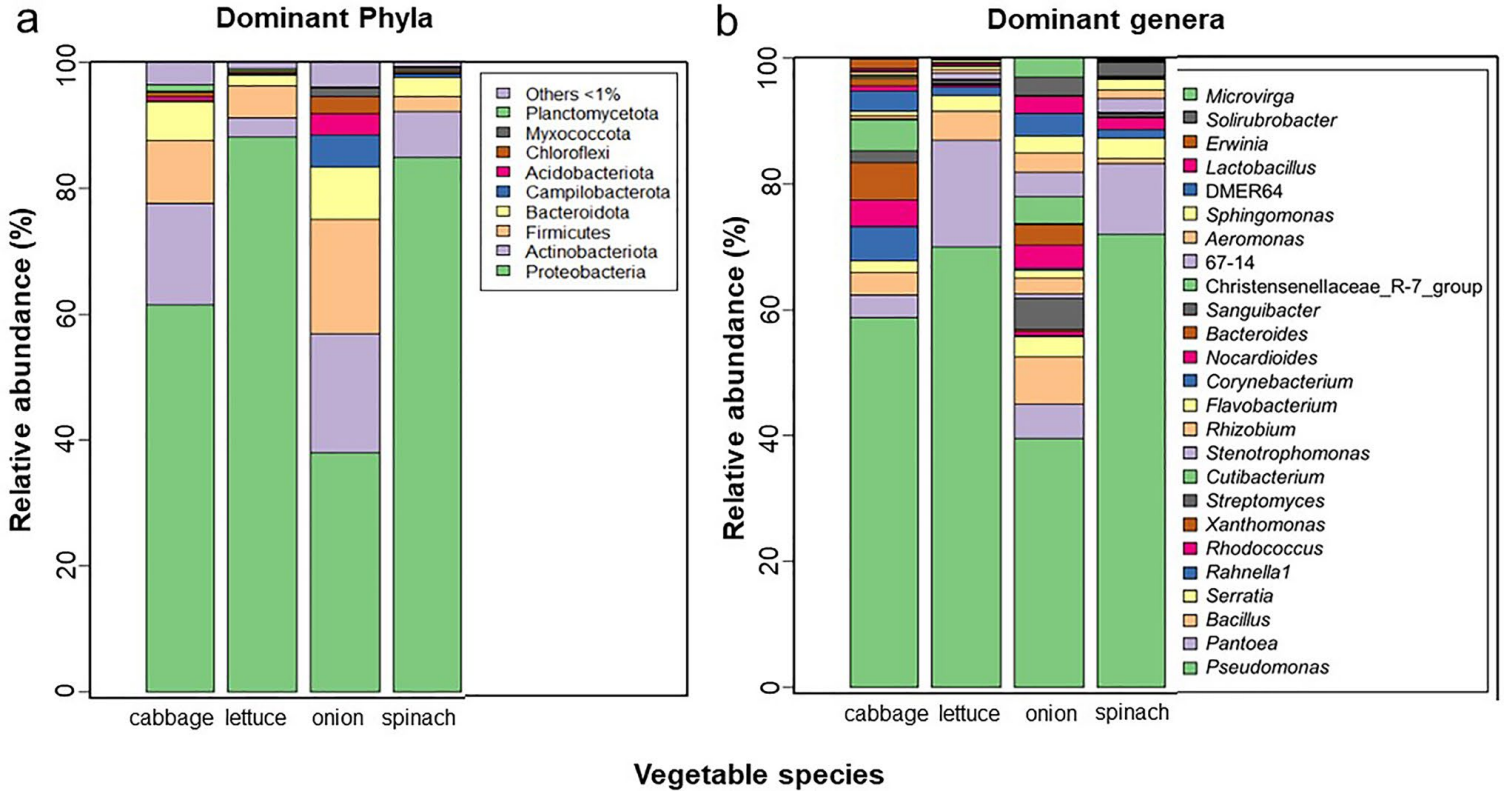
Mean relative abundance of dominant bacterial endophytes. This figure displays the mean relative abundance of dominant bacterial communities (those with > 1% average relative abundance) found in the four leafy vegetable species. Plot **a** shows the dominant phyla, and plot **b** shows the dominant genera. Taxonomic assignment identified Proteobacteria and Actinobacteriota as the most relatively abundant phyla, collectively constituting about 80% of the total bacterial community. At the genus level, *Pseudomonas*, *Pantoea*, *Bacillus*, *Serratia*, *Rahnella*1, *Rhodococcus*, *Xanthomonas*, and *Streptomyces* were among the most abundant across all vegetables, though their relative abundance varied by species. Specifically, *Pseudomonas* was highest in spinach, *Pantoea* in lettuce, *Bacillus* in onion, and *Rahnella*1 in cabbage. The root and organic farms exhibited higher diversity at the genus taxa level, with Proteobacteria being more abundant in root and organic farms and Actinobacteria higher in leaf and conventional farms

The filtered reads generated were 1,678,081, with 12,339 ± 1893 (mean ± SE) sequences per sample, which were clustered into 11,439 ASVs. The sequencing depth was confirmed adequate as indicated by the rarefaction curve (Supplementary Fig. 4) and the Good’s coverage index, which was 100% for all samples. Taxonomic assignment revealed the dominant ASVs (> 1% average relative abundance) belonged to 9 phyla and 25 genera (Fig. 4a, b), with Proteobacteria and Actinobacteriota being the most relatively abundant, representing about 80% of the total bacterial community at the phyla level. The genera *Pseudomonas*, *Pantoea*, *Bacillus*, *Serratia*, *Rahnella1*, *Rhodococcus, Xanthomonas* and *Streptomyces* were the most relatively abundant bacterial communities across the vegetables (Fig. 4b). However, their relative abundance varied, with onion, followed by cabbage having the highest bacterial community richness. In addition, *Pseudomonas, Pantoea, Bacillus* and *Rahnella1* had the highest relative abundances in spinach, lettuce, onion, and cabbage, respectively. Across the plant organ and fertilizer types, Proteobacteria was higher in root and organic farms compared to Actinobacteria, which is higher in leaf and conventional farms. The root and organic farms have a higher diversity at the genera taxa level (Supplementary Fig. 5).

**Fig. 5:**
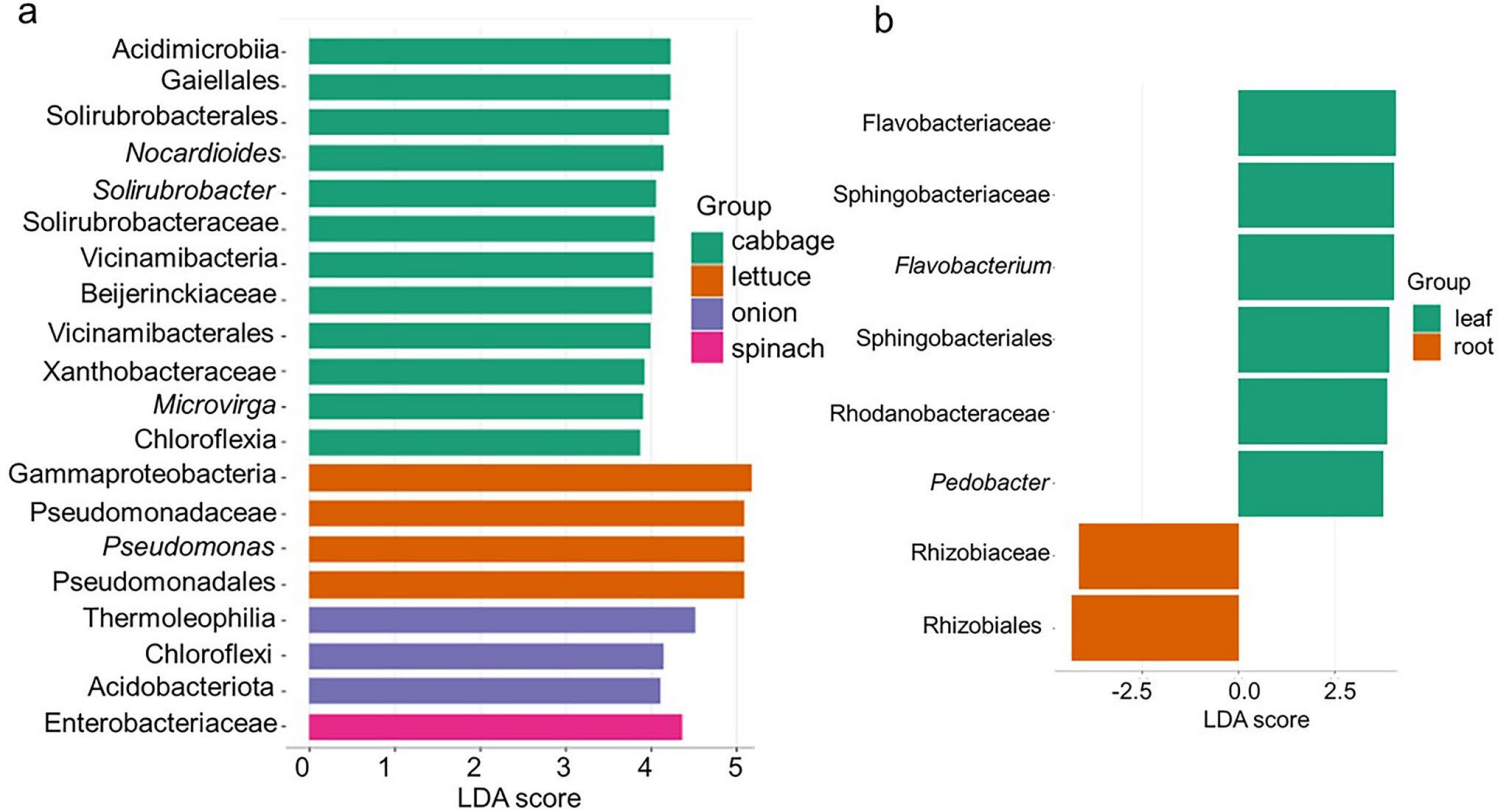
Significantly differentially abundant bacterial features by LEfSe analysis. This figure presents the significantly differentially abundant bacterial features identified across **a** plant species and **b** plant organ types using the linear discriminant analysis (LDA) effect size (LEfSe) method. For plant species, 131 discriminant features were initially found, with 33 remaining significant after *P* value adjustment (FDR-adjusted *Q* < 0.05, LDA score > 3.02). Key discriminant features included higher abundance of Galellales and Acidimicrobiia in cabbage, Gammaproteobacteria in lettuce, Thermoleophilla in onion, and the family Enterobacteriaceae in spinach. For plant organs, eight features were discriminant after *P* value adjustment (FDR-adjusted *Q* < 0.2, LDA score > 3.74), with Rhizobiales predominantly discriminant in the root and flavobacteriaceae in the leaf

Following a sample-wide LDA Effect Size (LEFSe) analysis, a total of 131 discriminant features were significant among the vegetable species, however, only 33 of these features were significant after P value adjustment (FDR-adjusted *Q* < 0.05, LDA score > 3.02) (Fig. 5a and Supplementary Table 6). No discriminant features were found significant for fertiliser practice, while eight features (Fig. 5b) were discriminant for plant organs after P value adjustment (FDR-adjusted *Q* < 0.2, LDA score > 3.74). The most discriminant features between plant species and organs are shown in Fig. 5a, b. Cabbage had a higher abundance of *Galellales* and *Acidimicrobiia*, while lettuce and onion had a higher abundance of Gammaproteobacteria and Thermoleophilla, respectively. Spinach was found to have the family Enterobacteriaceae as the only discriminant feature. *Rhizobiales* and flavobacteriaceae were predominantly discriminant in the root and leaf, respectively.

### Assessment of the diversity of unique and shared bacterial endophytes

**Fig. 6:**
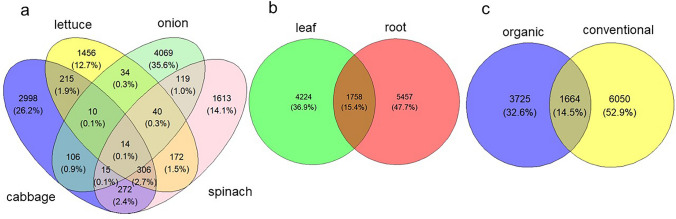
Distribution of unique and shared endophytic bacterial communities. This figure uses Venn diagrams to illustrate the distribution of unique and shared endophytic bacterial communities (ASVs) across **a** plant species (cabbage, lettuce, onion, and spinach), **b** plant organs (leaf and root), and **c** fertiliser types (organic and conventional). Onion exhibited the highest percentage of unique ASVs (35.6%). The highest percentage of shared bacterial communities (2.7%) occurred between cabbage and spinach. Only a small fraction (0.1%) of the total bacterial communities, belonging to Proteobacteria, Firmicutes, Bacteroidota, and Campylobacterota, were shared among all four vegetable species and may be considered the core microbiome. The root organ contained more unique ASVs than the leaf, with approximately 15% shared communities between them. Conventional farms (CF) showed a higher number of unique ASVs compared to organic farms (OF), although 14.5% of ASVs were shared between OF and CF farms. Classifiable bacterial communities shared among all vegetable species included genera such as *Pseudomonas*, *Bacillus*, and *Escherichia-Shigella*

Shared and unique bacterial communities vary among plant species, organs, and fertilizer types. The highest number of unique ASVs (35.6%) was observed in onion (Fig. 6a), followed by cabbage, spinach, and lettuce, while the highest percentage of shared bacterial communities (2.7%) occurred between cabbage and spinach. Only 0.1% of the total bacterial communities (belonging to Proteobacteria, Firmicutes, Bacteroidota and Campylobacterota) were shared among all the vegetable species (Fig. 6a) and may be regarded as the core microbiome of these vegetables. The root had more unique ASVs compared to the leaf, with about 15% of shared communities between the leaf and root (Fig. 6b). Although some ASVs (14.5%) were shared between OF and CF farms, a higher number of unique ASVs was observed in CF farms compared to OF farms vegetables (Fig. 6c). The unique and shared ASVs are taxonomically diverse, with no dominant taxa identified between the groups. The classifiable bacterial communities shared among all the vegetable species were 14 ASVs, which included *Pseudomonas, Bacillus, Escherichia-Shigella, Bacteroides, Proteiniphilum, Bacteroides, Dyadobacter, Clade_Ie,* and *Saccharofermentans*. Five other unclassified groups at the genus taxa level were identified in this group.

### Co-occurrence networks analysis, patterns, and keystone taxa

**Fig. 7:**
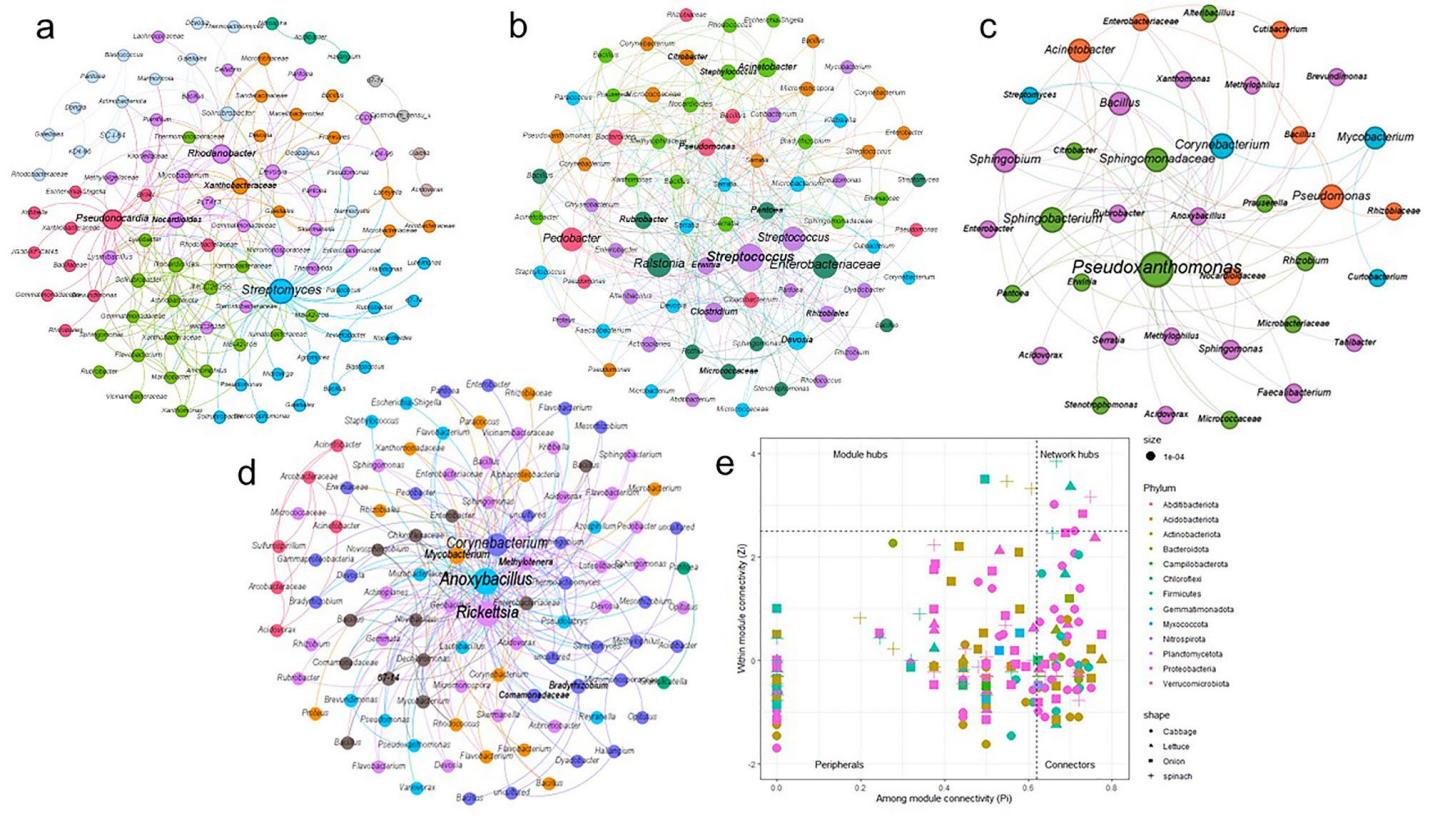
Microbial co-occurrence network analysis and keystone taxa identification. This figure presents the co-occurrence networks and node characterization, facilitating the detection of keystone taxa among bacterial communities across different plant species. The networks show high power-law distribution for lettuce, onion, and spinach (R2 > 0.75), suggesting non-random co-occurrence patterns. Plot **a** shows the network for onion, **b** for cabbage, **c** for lettuce, and **d** for spinach. Nodes represent bacterial species (specifically, genera) and are colored based on their community modularity class. The size of each node reflects its degree of distribution. Edges represent network connections indicating significant (FDR-adjusted *P* < 0.01) associations between nodes, with edge size corresponding to the weight of the association. Network modularity indices ranged from 0.235 to 0.396, suggesting relative homogeneity, with onion having the highest modularity (0.396), implying a potentially more stable community structure. Interaction patterns varied, with spinach showing the most positive associations, while onion and cabbage primarily exhibited negative links. A high proportion of negative associations suggests competition among endophytes. Module classification **e** categorizes nodes into peripherals (Zi < 2.5; Pi < 0.62), connectors (Zi < 2.5; Pi > 0.62), module hubs (Zi > 2.5; Pi < 0.62), and network hubs (Zi > 2.5; Pi > 0.62). More than 60% of the core genera were classified as peripherals. Key ‘network hub’ taxa identified for the plant species factor included Anoxybacillus, Serratia, Streptomyces, Pseudonocardia, Rickettsia, and Methylotenera. ‘Module hub’ taxa included Solirubrobacter in the onion network and Corynebacterium and Mycobacterium in the spinach network

The networks showed high power-law distribution for plant species (lettuce R2 = 0.86; *P* < 0.01, onion R2 = 0.91; *P* < 0.05, spinach R2 = 0.75; *P* < 0.01), except for (cabbage R2 = 0.27; *P* < 0.05). The high R2 values and structural properties for the empirical networks compared to random networks (Table 3) suggest that the co-occurrence patterns are non-random. The empirical networks for the plant species had 38 to 103 nodes (corresponding to genera), indicating bacterial interactions, with each node having at least 1–21 edges. The network modularity index ranges from 0.235 to 0.396, suggesting a relative homogeneity of the bacterial community between vegetable species and fertiliser practice; values > 0.4 suggest a modular structure (Newman 2006). Spinach had the highest number of positive associations between microbial communities while onion and cabbage only showed negative links (Fig. 7a–d). The average network path distance between paired nodes was 2.42 edges across the plant species. Onion had the highest average path distance of 2.97, followed by spinach, cabbage, and lettuce. The node clustering coefficient measuring how closely connected nodes are in a network ranged from 0.03 to 0.19 and was highest in lettuce compared to other plant species.

**Table 3:** Structural properties of empirical and random networks between bacterial communities of vegetables

Network indexes	Cabbage		Spinach		Onion		Lettuce	
	ENI	100 RNI	ENI	100 RNI	ENI	100 RNI	ENI	100 RNI
Average clustering coefficient (avgCC)	0.076	0.171 ± 0.016	0.188	0.441 ± 0.025	0.031	0.164 ± 0.022	0.194	0.295 ± 0.040
Average path distance (GD)	2.227	2.253 ± 0.016	2.316	2.363 ± 0.026	2.974	2.846 ± 0.052	2.162	2.209 ± 0.039
Geodesic efficiency (E)	0.501	0.497 ± 0.002	0.473	0.461 ± 0.003	0.396	0.396 ± 0.005	0.514	0.509 ± 0.005
Harmonic geodesic distance (HD)	1.997	2.012 ± 0.009	2.113	2.170 ± 0.016	2.524	2.525 ± 0.032	1.945	1.967 ± 0.019
Centralization of degree (CD)	0.209	0.209 ± 0.000	0.476	0.476 ± 0.000	0.355	0.355 ± 0.000	0.586	0.586 ± 0.000
Centralization of betweenness (CB)	0.101	0.093 ± 0.013	0.232	0.310 ± 0.018	0.360	0.375 ± 0.024	0.515	0.504 ± 0.029
Centralization of stress centrality (CS)	0.444	0.390 ± 0.046	0.871	0.741 ± 0.047	1.307	0.954 ± 0.085	1.009	0.948 ± 0.120
Centralization of eigenvector centrality (CE)	0.137	0.164 ± 0.010	0.308	0.321 ± 0.007	0.310	0.361 ± 0.017	0.357	0.357 ± 0.014
Density (D)	0.112	0.112 ± 0.000	0.054	0.054 ± 0.000	0.044	0.044 ± 0.000	0.121	0.121 ± 0.000
Reciprocity	1	1.000 ± 0.000	1	1.000 ± 0.000	1	1.000 ± 0.000	1	1.000 ± 0.000
Transitivity (Trans)	0.051	0.156 ± 0.010	0.064	0.136 ± 0.007	0.026	0.139 ± 0.013	0.123	0.190 ± 0.022
Connectedness (Con)	1	1.000 ± 0.000	0.850	0.991 ± 0.017	0.869	0.965 ± 0.033	1	0.999 ± 0.010
Efficiency	0.900	0.900 ± 0.000	0.947	0.955 ± 0.001	0.960	0.964 ± 0.001	0.903	0.903 ± 0.001
Hierarchy	0	0.000 ± 0.000	0	0.000 ± 0.000	0	0.000 ± 0.000	0	0.000 ± 0.000
Lubness	1	1.000 ± 0.000	1	1.000 ± 0.000	1	1.000 ± 0.000	1	1.000 ± 0.000
Modularity(fast_greedy)	0.235	0.244 ± 0.009	0.313	0.287 ± 0.008	0.396	0.377 ± 0.009	0.259	0.283 ± 0.016

The networks showed low modularity, with onion (8 modules) having the highest number of modules followed by spinach, cabbage, and lettuce (Table 3). More than 45% of the nodes (> 1% relative abundance) were grouped as peripherals and less than 53% were categorized into connectors in each plant species network. The number of nodes grouped into network and module hubs was relatively small, with the highest percentage found to be only 3% and 2% in spinach, respectively. The lettuce network nodes are closer and more connected to their neighbors as revealed by their higher clustering coefficient and geodesic efficiency compared to other networks (Table 3).

Of all the plant species networks, onion has a more stable community due to its high modularity values compared to random chance (Fig. 7a). More than 60% of the core genera belonged to the generalist (peripheral) (Fig. 7e), with the dominant being *Pseudomonas*, *Bacillus, Agromyces* and *Nocardioides* as well as other unclassified genera in the phyla Proteobacteria, Firmicutes, and Bacteroidetes. Six bacterial communities: *Anoxybacillus, Serratia, Streptomyces, Pseudonocardia, Rickettsia,* and *Methylotenera* were identified as “network hub” taxa for the plant species factor. The module hub taxa are *Solirubrobacter* found in the onion network and *Corynebacterium* and *Mycobacterium* found in the spinach network (Fig. 7e).

### Predicted functional structure and differentially abundant metabolic pathways

The PICRUSt2 analysis revealed that about 98% of the total ASVs successfully mapped with the Kyoto Encyclopaedia Genes and Genomes (KEGG), and 1.6% of the sequences having above the maximum NSTI cut-off of 2 were expunged. A total of 8,185 KO terms, 447 metabolic pathways, and 2490 enzyme classification (EC) metabolic functions with species contributions were inferred from all ASVs. The pathways grouped into 39 secondary superclass 2 with the most abundant pathways (> 5% relative abundance) being cofactor carrier and vitamin (14.3%), aromatic compound degradation (10%), amino acid biosynthesis (8.7%), nucleoside and nucleotide degradation (6.7%), carbohydrate biosynthesis (5.4%), carbohydrate degradation (5.4) and fatty acid and lipid (5.2%).

**Fig. 8:**
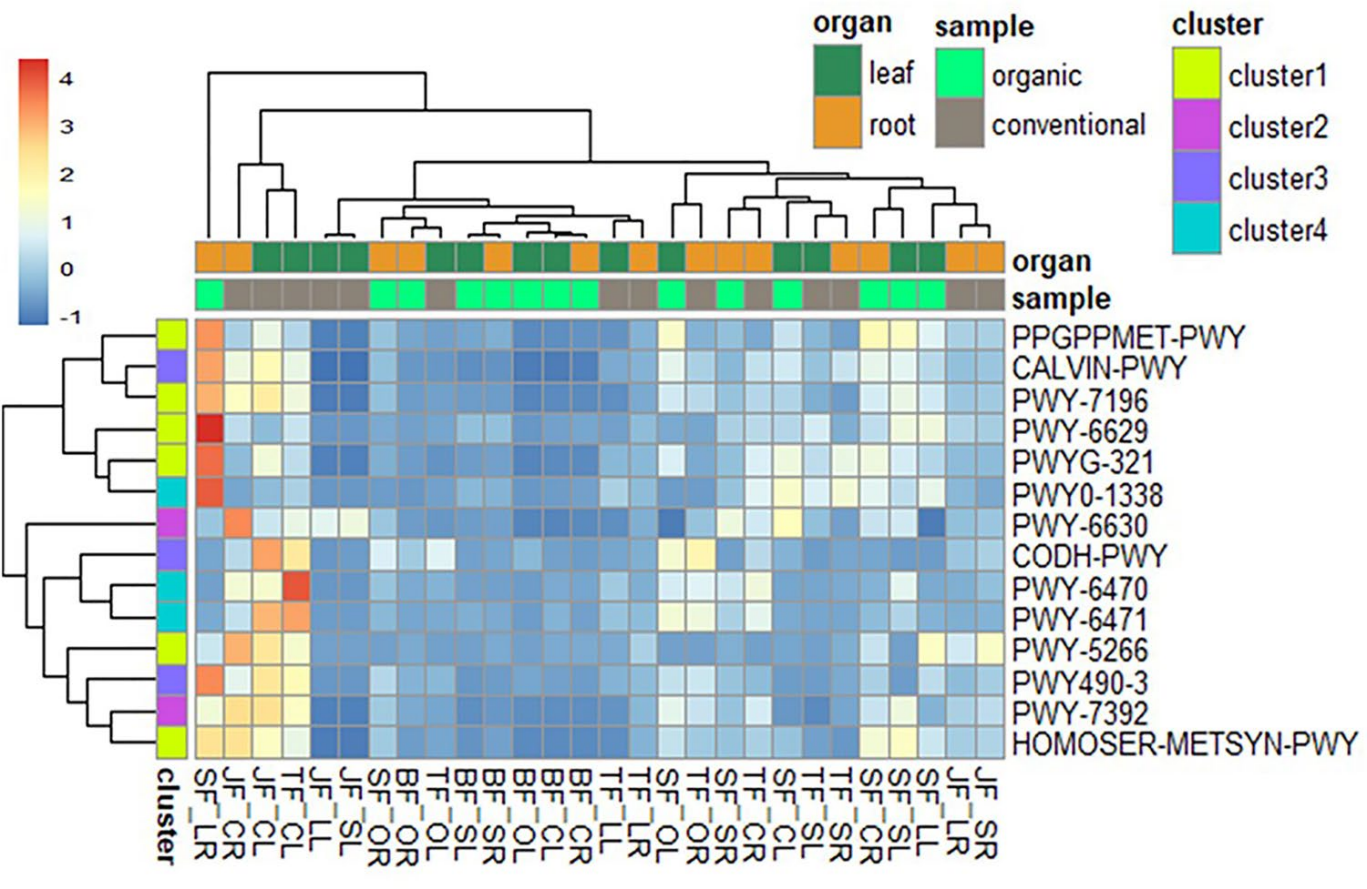
Relative abundance of selected economically important metabolic pathways. This figure illustrates the relative abundance of selected metabolic pathways predicted from the endophytic bacterial communities across fertilizer type, plant species, and organ types, based on PICRUSt2 analysis. The pathways are grouped into four clusters based on their potential functions: cluster 1 (bacterial virulence or pathogenicity), cluster 2 (biotechnological importance), cluster 3 (ecological functions), and cluster 4 (bacterial antibiotic resistance). The PICRUSt2 analysis inferred 8185 KO terms, 447 metabolic pathways, and 2490 enzyme classification (EC) metabolic functions

Important pathways such as CALVIN-PWY and CODH-PWY (involved in CO_2_ fixation) and PWY490-3 (nitrate reduction) contributing to ecological functions were not particularly different across the OF or CF farms (Fig. 8). However, CALVIN-PWY and PWY490-3 were highly prevalent among bacterial endophytes in OF farm SF under lettuce production while CODH-PWY was predominantly abundant in CF farm JF under cabbage production (Fig. 8). Pathways associated with bacterial antibiotic resistance, including PWY-6470; β-lactam resistance and PWY-6471; peptidoglycan biosynthesis IV were predominant in CF compared to OF farms. whereas PWY0-1338; polymyxin resistance was higher in bacterial endophytes from vegetables in OF compared to CF farm (Fig. 8). The pathways promoting bacterial virulence or pathogenicity such as PWY-6629; l-tryptophan biosynthesis, PPGPPMET-PWY; guanosine 3′-diphosphate 5′-diphosphate biosynthesis, PWYG-321; mycolate biosynthesis, and PWY-7196; pyrimidine ribonucleosides salvage were highly predicted in OF farms, with farm SF having the highest abundance.

The PWY-5266; p-cymene degradation was more abundant in CF compared to the OF farm and highly prevalent in bacterial endophytes from farm JF. The abundance of HOMOSER-METSYN-PWY; l-methionine biosynthesis I was comparatively similar across the farms. These pathways influence bacterial pathogenicity. Pathways of biotechnological importance such as PWY-7392; Taxadiene biosynthesis and PWY-6630; l-tyrosine biosynthesis were highly predicted in bacterial endophytes of vegetables from CF farms (Fig. 8). Contrary to the fertilizer type factor, the organ types significantly (*P* < 0.05) affected the pathway's abundance. Only PWY0-1338, CODH-PWY, PWY490-3, and PWY-5266 were significantly affected by the plant types and the interaction between plant and fertilizer type significantly (ANOVA, *P* < 0.05) influenced the selected pathways.

**Fig. 9:**
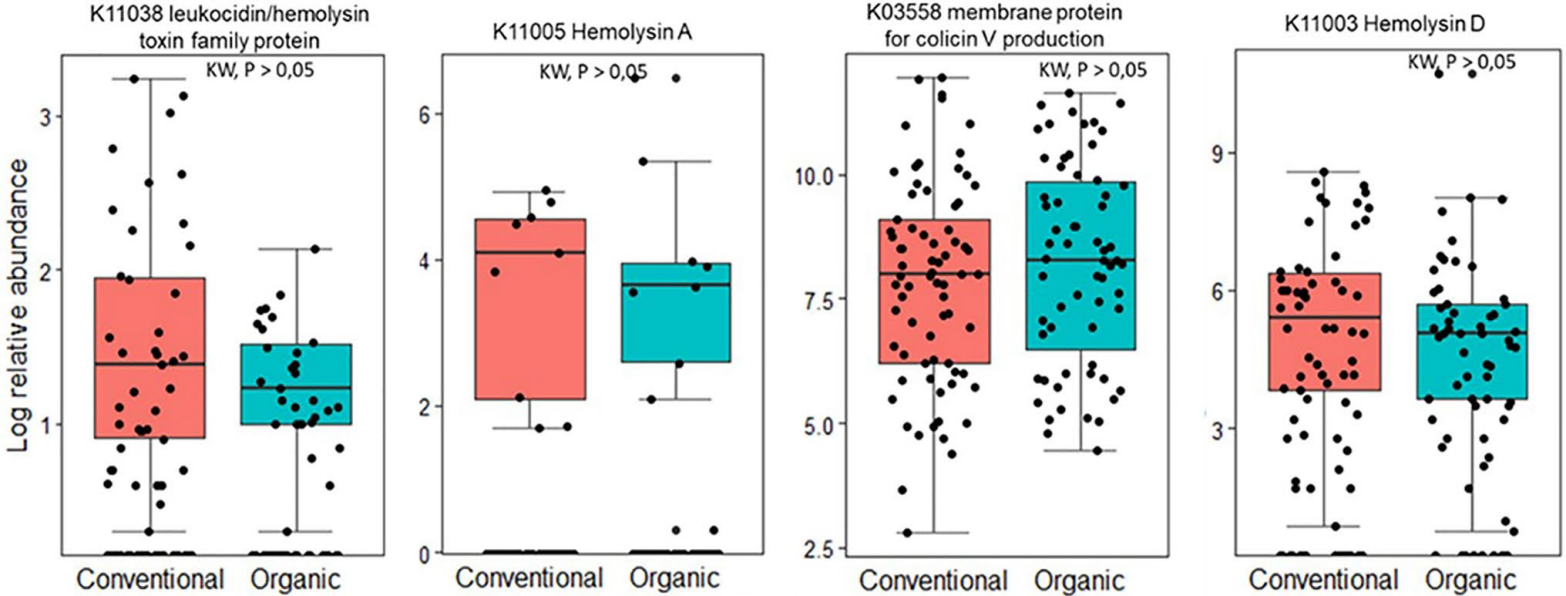
Abundance of selected toxin and hemolytic genes. This figure shows the log relative abundance of selected toxin and hemolytic genes within the endophytic bacterial communities of vegetables cultivated under organic and conventional fertilizers. The genes assessed include leucocidin and hemolysin toxin family protein (K11038), membrane protein required for colicin production (K03558), hemolysin A (K11005), and hemolysin D (K11003). These genes are assessed for their potential to affect plant nutritional values. While these genes were not significantly different across the fertiliser practices (Kruskal–Wallis test), their mean abundance varied. Bacterial communities from vegetables cultivated under conventional farms (CF) generally showed higher mean abundance for K11038, K11005, and K11003 compared to those from organic farms (OF)

Toxin-producing genes, such as leucocidin and hemolysin toxin family protein (K11038), membrane protein required for colicin production (K03558), and hemolysin A (K11005), and D (K11003), which potentially affect plant nutritional values were assessed using their abundance across fertilizer practice (Fig. 9). Though these genes were not significantly different across the fertiliser practice, their mean abundance differed. For K11038, K11005 and K11003, the bacterial communities from vegetables cultivated under CF farms had higher abundance compared to that of OF farms.

**Fig. 10:**
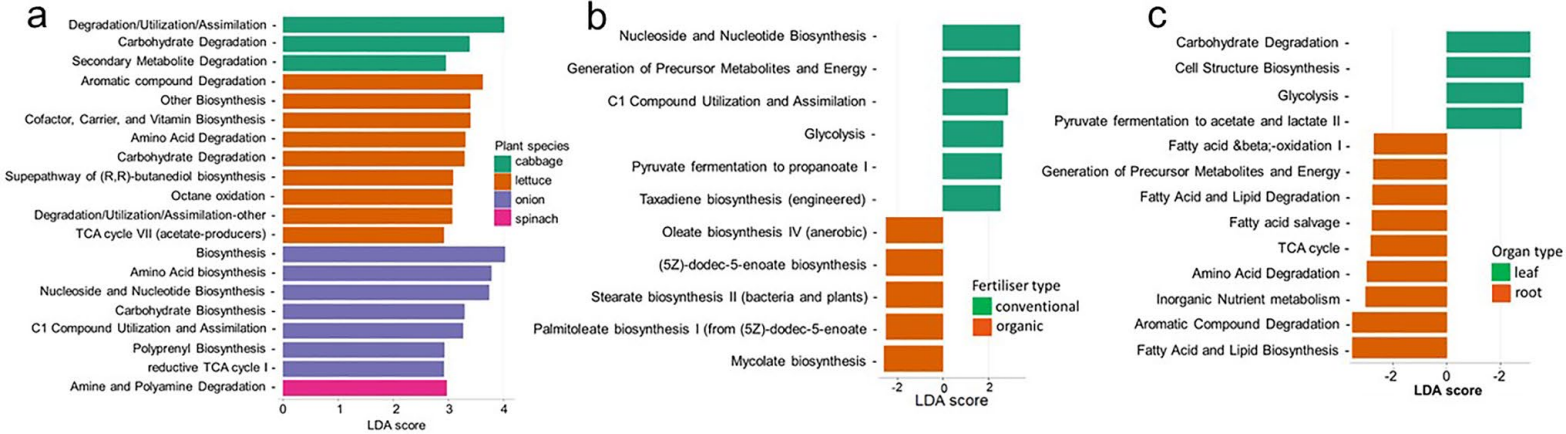
Distribution of discriminant metabolic pathways. This figure displays the distribution of discriminant metabolic pathways identified across the fixed factors: **a** plant species, **b** fertilizer type, and **c** organ type. Pathways were identified as significant after *P* value adjustment (FDR-adjusted *Q* < 0.05, LDA score > 2.50 for species; FDR-adjusted *Q* < 0.2, LDA score > 2.4 for organs and fertilizer types). For plant species (**a**), degradation/utilization/assimilation pathways were highly discriminant in cabbage, aromatic compound degradation in lettuce, amino acid biosynthesis in onion, and amine and polyamine degradation in spinach. For fertiliser type (**b**), nucleoside and nucleotide biosynthesis and generation of precursor metabolite and energy were more discriminant in conventional farms (CF), while Mycolate, palmitoleate, and oleate biosynthesis were highly discriminant in organic farms (OF). For organ type (**c**), glycolysis, cell structure biosynthesis, and carbohydrate degradation were more discriminant in leaves, while aromatic, amino acid, fatty acid and lipid degradation/biosynthesis, and inorganic nutrient metabolism were highly discriminant in roots

Degradation/utilization/assimilation pathway was highly discriminant among communities from cabbage while aromatic compound degradation, amino acid biosynthesis and amine and polyamine degradation were predominantly discriminant in lettuce, onion, and spinach, respectively (Fig. 10a). Fertilizer type factor may have influenced the abundance of nucleoside and nucleotide biosynthesis and generation of precursor metabolite and energy in CF farms. Mycolate, palmitoleate and oleate biosynthesis were highly discriminant in the OF farms (Fig. 10b). In addition, glycolysis, cell structure biosynthesis and carbohydrate degradation were more discriminant in the leaf while aromatic, amino acid, fatty acid and lipid compound degradation, fatty acid and lipid biosynthesis and inorganic nutrient metabolism were highly discriminant in the root Fig. 10c.

### Relationship between vegetable nutrient content, endophytic bacterial community and predicted metabolic pathways

**Fig. 11:**
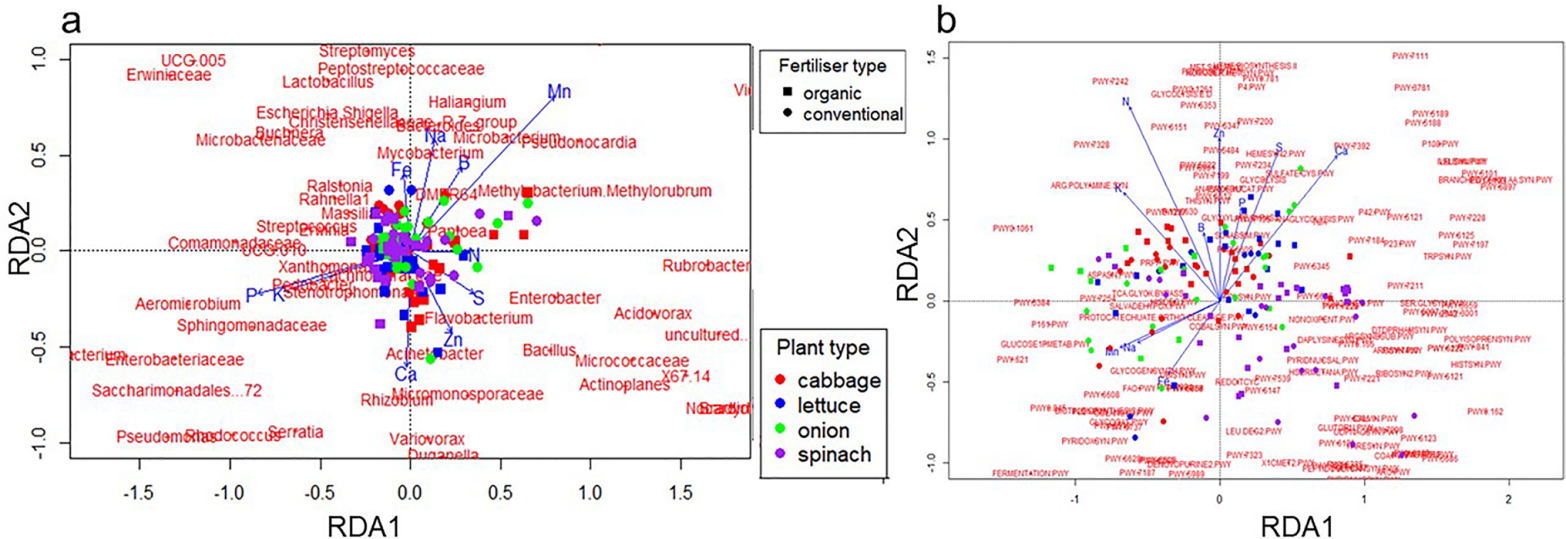
Relationship between vegetable nutrient content and endophytic bacterial communities/metabolic pathways via redundancy analysis (RDA). This figure utilizes redundancy analysis (RDA) to illustrate the correlation between vegetable nutrient content and **a** bacterial endophyte communities and **b** predicted metabolic pathways across plant species and fertilizer types. The explanatory variables are the physicochemical parameters measured as nutrient content (Mn, Ca, Zn, Fe, Na, B, P, K). The RDA analysis revealed that plant nutrient content explains up to 15.7% of the total variance in the bacterial community composition (**a**) and 16.9% of the total variance in the predicted metabolic pathways (**b**). The overall model for the triplot was statistically significant (ANOVA, *P* = 0.001). Among the assessed nutrients, P, Ca, S, Fe, and B significantly impacted the microbial community composition, while N, K, Na, and Zn significantly influenced the metabolic pathways. A significant positive correlation was found between overall vegetable nutrient content and the abundance of predicted metabolic pathways. Furthermore, alpha diversity metrics (Shannon, Chao1, Pielou’s evenness) were significantly correlated with nutrient content

The vegetable nutritional content had a significant (*r* = 0.0798, *P* < 0.03) and positively strong correlation with the predicted metabolic pathway abundance. The alpha diversity measured, including Shannon, Chao1 and Pielou’s evenness, were significantly (*r* = 0.053, *P* = 0.0348) correlated to the nutrient content. In addition, the RDA analysis suggests that the plant nutrient contents explain up to 15.7% and 16.9% of the total variance in the bacterial community composition (Fig. 11a) and the predicted metabolic pathways (Fig. 11b), respectively, as revealed by the significant (ANOVA, *P* = 0.001) model for the triplot. Among the explanatory variables assessed, P, Ca, S. Fe and B significantly impact the microbial community composition, while N, K, Na, and Zn, significantly influence the metabolic pathways. The Shannon-Weiner index had a negatively significant relationship with K in the OF farm and B in the CF farm (Supplementary Fig. 6). Similarly, a significant negative relationship was observed for Chao1 and N, K, Mg, S, Cu and Zn in OF farms, and only Mn, Na, Fe and B in CF farms (Supplementary Fig. 7). A positive and significant association was observed between Pielou’s evenness and Ca, Mg, S and B for the OF farm vegetables (Supplementary Fig. 8).

## Discussion

Plant microbiomes are well-studied for their ecological importance, especially as they influence plant health and productivity (Adeleke et al. 2019; Ameen et al. 2024), creating awareness among ecologists to further comprehend the nexus between plant microbiomes and plant nutritional quality. This study focused on evaluating the diversity and potential functionalities of endophytic bacterial communities residing within four common leafy vegetables: *Brassica oleracea* (cabbage), *Lactuca sativa* (lettuce), *Allium cepa* (onion), and *Spinacia oleracea* (spinach). The investigation specifically determined how different cultivation practices, namely organic and conventional farming systems, as well as intrinsic plant factors such as species and organ (leaf versus root), influence these microbial communities. Utilizing high-throughput 16S rRNA gene sequencing, coupled with the PICRUSt2 pipeline for functional prediction, the research sought to uncover potential implications of these microbial variations for both plant microbiological quality and, ultimately, human health, particularly considering vegetables often consumed raw.

### Influence of plant species, organ, and fertilizer type on endophytic bacterial communities

A pivotal finding in this study highlighted the significant influence of plant species and organ type on the composition of endophytic bacterial communities. Beta diversity analysis indicated a substantial overlap among communities found in cabbage, lettuce, and spinach (Fig. 3a, b). However, onion communities exhibited a distinct clustering pattern, significantly differing from the others, suggesting that this plant species harbors a unique associated microbiome. Although, this aligns with research trend in this field, emphasizing the critical role of plant species and organs in shaping microbial endophyte diversity (Afzal et al. 2019; Adeleke et al. 2019), it is also an interesting area of future research to investigate the drivers of onion’s endophytic community. Alpha diversity metrics (Fig. 2a–d), including Chao1, Shannon-Weiner, Pielou's evenness and Simpson, further supported this variability, showing significant differences across vegetable species and organs. Bacterial endophyte abundance and richness were notably lower in the leaves compared to the roots. This lower microbial complexity in the phyllosphere (leaves) compared to below-ground ecosystems (rhizosphere soil) is well-reported in other studies (Guo et al. 2021; Dastogeer et al. 2020). The higher diversity in roots may be attributed to the dense and complex microbial communities present in soil and the rhizosphere, offering greater proximity for potential endophytes to colonize roots (Ahkami et al. 2017). Furthermore, the phyllosphere is less protected from daily and seasonal environmental variations such as heat, moisture, radiation, rainfall, and winds, which affect plant physiologic activities like respiration and photosynthesis (Dastogeer et al. 2020). These activities can drive endophyte colonization and influence microbial adaptation strategies, contributing to the differential diversity and compositional profiles observed across plant organs. Furthermore, it is pertinent to mention that statistical limitations may critically impact the interpretation of differential abundance of bacteria in metabarcoding data, affecting analysis of alpha and beta diversity (Gleason et al. 2023; Roche and Mukherjee 2022). These challenges stem from issues such as biases in data quality (e.g., PCR errors, low specificity), insufficient sampling efforts, and methodological problems such as zero-inflation and the trade-offs between sensitivity and specificity in models (Gleason et al. 2023; Roche and Mukherjee 2022; Jurburg et al. 2022). Consequently, these limitations can distort observed microbial diversity, result in alarmingly high false positive rates in simulated datasets, skew diversity estimates, and mean that real datasets may not achieve predicted sensitivity levels. Although, we took all the necessary measures to undertake both our wet and dry lab activities for this study, it is not uncommon, for any of such factors to have played a role.

For fertiliser management type, comparing OF versus CF, also significantly impacted bacterial diversity measures. Interestingly, the results revealed higher bacterial diversity on CF compared to OF. This difference extended to phylogenetic diversity, which was also significantly higher in conventional farms. This finding contrasts with some other studies which reported higher endophytic bacterial diversity in crops cultivated under OF farms (Koishi et al. 2020; Peltoniemi et al. 2021). Meanwhile, another study reported lower bacterial endophyte diversity in lettuce cultivated under OF farms compared to CF (Zhu et al. 2017). However, despite the significant effect of fertilizer type on overall diversity, the study found no differentially abundant bacterial features that were statistically significant for fertiliser practice. This suggests that while fertilizer type influences overall richness and evenness, it may not drive distinct taxonomic differences in the most abundant or differentiating groups as strongly as plant species or organ type. This conclusion is supported by PERMANOVA analysis, where the significant main effects on bacterial community composition were primarily attributable to the plant species type itself, rather than just variations in dispersion (PERMANOVA, *P* > 0.05, Supplementary Table 5). The results regarding fertiliser-type clustering revealed no distinct clustering based on this factor (Fig. 3). Various factors interact in complex ways to influence plant microbial community compositions, highlighting the necessity of studying microbiomes under the influence of multiple ecological and agronomic factors to gain a more comprehensive understanding. However, it is often difficult to combine several factors at a single site, suggesting that no specific set of factors can definitively explain how bacterial communities of a plant species vary in composition and diversity.

### Dominant taxa and core microbiome

At the broadest level, the study identified Proteobacteria and Actinobacteriota as the most dominant phyla**,** collectively representing approximately 80% of the total bacterial community (Fig. 4). Proteobacteria were found to dominate the phyllosphere bacterial community. This observation aligns with findings from several other studies (Chimwamurombe et al. 2016; Guo et al. 2021). However, certain studies have reported Firmicutes as the most abundant phylum in vegetables, suggesting that ecological factors such as location and crop types may be influential (Xia et al. 2015; Khalaf and Raizada 2016). Organ type and farming system influenced the distribution of these dominant phyla. Proteobacteria were more abundant in root samples and organic farms, while Actinobacteria were more prevalent in leaves and conventional farms (Fig. 4).

At the genus level, the root and organic farms also showed higher diversity (Fig. 4). *Pseudomonas*, *Pantoea*, *Bacillus*, *Serratia*, *Rahnella1*, *Rhodococcus*, *Xanthomonas*, and *Streptomyces* were among the most abundant across all tested vegetables. The relative abundance of these dominant genera varied depending on the specific vegetable type. For instance, *Pseudomonas* was most abundant in spinach, *Pantoea* in lettuce, *Bacillus* in onion, and *Rahnella*1 in cabbage. The dominance of genera like *Pseudomonas* and *Bacillus* is particularly noteworthy, as these taxa are known to include plant growth-promoting bacteria (PGP) with potential agroecological roles (Garritano et al. 2022; Hudson 2023). These roles can include producing indole acetic acid, which promotes plant growth, and solubilising phosphate, making it available to the plant (Wang et al. 2016; Raimi and Adeleke 2023). Other studies have also isolated *Bacillus*, *Pseudomonas*, and *Pantoea* from lettuce plants (Hou et al. 2013; Raimi and Adeleke 2023).

An analysis of unique and shared bacterial endophytes provided further insights into the community structure across the fixed factors. Onion harbored the highest number of unique Amplicon Sequence Variants (ASVs), reinforcing the finding of its distinct community structure (Figs. 3, 6a). The root organ also contained more unique ASVs compared to the leaf (Fig. 6b). Interestingly, conventional farms exhibited a higher number of unique ASVs compared to organic farms (Fig. 6c). Only a small fraction (0.1%) of the total bacterial communities were shared among all four vegetable species, constituting a potential core microbiome (Fig. 6a). This core microbiome included genera such as *Pseudomonas*, *Bacillus*, and *Escherichia*-*Shigella*. The presence of these taxa consistently across vegetable types regardless of species or farm management suggests they may perform key functions impacting host physiologic performance. Consistent with other studies, bacterial genera such as *Pseudomonas*, *Bacillus*, *Pantoea*, *Serratia*, and *Rahnella*1 were found to constitute the core bacterial communities (Bulgarelli et al. 2013; Chang et al. 2022; Kgoale et al. 2023).

### Microbial interactions and keystone taxa

The co-occurrence network analysis revealed that the observed bacterial community patterns were non-random, indicating meaningful interactions among taxa (Fig. 7). The networks generally exhibited low modularity, suggesting a relative homogeneity of the bacterial community within the tested variables. However, onion’s network displayed the highest modularity, implying a potentially more stable community structure compared to random chance (Fig. 7e). Different interaction patterns were observed across vegetable species. Spinach showed the highest number of positive associations, while onion and cabbage exhibited only negative links. The plant species was found to affect the vegetable endophytic bacterial association, with a high proportion of negative associations among the communities observed (Fig. 7a–d). This high proportion of negative associations suggests the impact of competition among the endophytic communities. Competition may have developed due to nutrient demand caused by functional redundancy (Sasaki et al. 2015). According to Deng et al. (2012) and Zhou et al. (2022), multiple factors drive the relationship between specific agricultural practices and network topologic features. They found that organic fertilizers caused an increase in the subnetwork size in the subsoil, facilitating the migration of microbes across soil profiles.

Identifying keystone taxa—defined as closely associated taxa with a considerable impact on microbial structure and function—is crucial for understanding plant–microbe interactions. The study identified several ‘network hub’ taxa associated with the plant species factor. These included *Anoxybacillus*, *Serratia*, *Streptomyces*, *Pseudonocardia*, *Rickettsia*, and *Methylotenera*. Module hub taxa specifically identified within the network analyses included *Solirubrobacter* in the onion network and *Corynebacterium* and *Mycobacterium* in the spinach network. *Solirubrobacter* was identified as a module hub taxon in onion and has a unique potential for establishing a niche for the survival of that bacterial community group. *Solirubrobacter* is known as a keystone taxon in soil ecosystems (Xun et al. 2021). These identified keystone taxa, despite possibly not being the most abundant members of the community (as most core genera were grouped as peripherals), are predicted to play significant roles in shaping the endophytic community structure. This could potentially influence plant nutritional quality. The identification of ‘generalists’ using the among/within module connectivity approach provided a comprehensive insight into microbial interactions, presenting a more representative network modularity and highlighting the role of nodes (taxa) among and within the overall structure.

### Predicted functional capabilities of endophytic bacteria

Based on the functional profiling of endophytic bacteria using the PICRUSt2 pipeline, the study revealed a wide array of predicted capabilities, including cofactor and vitamin biosynthesis, aromatic compound degradation, and amino acid biosynthesis, consistent with plant-associated bacteria. Significant differences were observed based on farm management and organ type. Endophytes from organic farms showed a higher prediction of pathways potentially promoting bacterial virulence or pathogenicity, such as guanosine 3′-diphosphate 5′-diphosphate, mycolate biosynthesis, and pyrimidine ribonucleosides salvage. In contrast, endophytes from conventional farms were predominant in pathways involved in physiologic functions potentially impacting the human immune system, like taxadiene biosynthesis and l-isoleucine biosynthesis. CFs, with higher levels of various nutrients (P, K, Ca, Fe, S, Zn), were also associated with higher relative abundance of biosynthesis pathways, including cell structure, vitamin, fatty acid and lipid, and nucleoside and nucleotide biosynthesis. This suggests farming practices influence endophytic functional potential, potentially impacting plant quality and human health upon consumption (Quémard 2016; Shaun Lott 2020; Mu et al. 2023).

Organ type also significantly influenced pathway abundance. Carbohydrate degradation pathways were more prevalent in leaves, likely due to the abundance of organic substrates like sugar and protein synthesized there (Figs. 8, 10). Conversely, pathways for aromatic compound, amino acid, fatty acid, and lipid degradation and biosynthesis, along with inorganic nutrient metabolism, were highly discriminant in roots (Figs. 8, 10). The high abundance of nutrient metabolism and degradation pathways in roots emphasizes their role, including root endophytes, in supplying nutrients to the upper plant parts (Agrawal et al. 2019; Raes et al. 2021). Endophytes from genera like *Bacillus*, *Enterobacter*, and *Pseudomonas* contribute to ecosystem functions; those with CALVIN and CODH pathways convert CO_2_ to organic matter, while those with nitrate reduction pathways contribute to the nitrogen cycle (Sparacino-Watkins et al. 2014; Garritano et al. 2022; Hudson 2023). Pathways contributing to bioactive compounds like taxadiene and l-tyrosine biosynthesis, suggesting therapeutic potential, were also higher in conventional farms (Flores-Bustamante et al. 2010; Juminaga et al. 2012; Raimi and Adeleke 2021). Understanding agronomic practices is key to enhancing the production of such important pathways.

### Relationship between nutrients, microbiome, and function

Exploring the interrelationship between vegetable nutrient content, the endophytic bacterial community, and its predicted metabolic functions provided crucial insights. A significant positive correlation was found between vegetable nutrient content and the abundance of predicted metabolic pathways. Furthermore, alpha diversity measures (Shannon, Chao1, Pielou’s evenness) were significantly correlated with nutrient content. Redundancy analysis (RDA) indicated that plant nutrient ted metabolic pathways (16.9%). Specific nutrients such as Phosphorus (P), Calcium (Ca), Sulfur (S), Iron (Fe), and Boron (B) were identified as having a significant impact on microbial community composition. Meanwhile, Nitrogen (N), Potassium (K), Sodium (Na), and Zinc (Zn) were found to significantly influence metabolic pathways. These correlations suggest a complex interaction where the plant's nutritional status may select for or influence the endophytic microbiome. This microbiome, in turn, possesses metabolic capabilities potentially contributing to plant quality and, consequently, the nutritional value obtained by consumers. Vegetable nutrient content was found to differ across crop types and fertilizer types (Chaudhari 2023; Tian et al. 2023). These differences are related to soil fertility, climate, and plant types. Biologic, chemical, and physical factors not only affect the nutrient composition of plant tissues but also influence associated microbiomes and their functions (Brown et al. 2020; Tian et al. 2023; Bhardwaj et al. 2024; Ahkami et al. 2017; Wipf et al. 2021).

### Potential implications for human health and microbiological quality

Consuming raw vegetables introduces millions of microbial cells into the human gut, potentially affecting human health due to certain endophyte functional profiles. This study identified pathways, such as PPGPPMET, PWY-6629, HOMOSER-METSYN-PWY, and PWYG-321(Fig. 8), that enhance microbial virulence and bacterial pathogenicity, which are of great human health concern. A key challenge is that bacterial endophytes cannot be eliminated by surface washing, disinfecting, or even cooking, meaning they can integrate into the human gut microbiome and potentially serve as a source of opportunistic pathogens. Growing concerns about vegetable safety highlight that potential sources of pathogens and ARGs include organic substrates like animal manure used as fertilizers and contaminated water sources. For instance, lettuce cultivated under organic farming was reported to have a significantly higher abundance of antibiotic-resistant bacteria and genes compared to conventional practice (Hölzel et al. 2018; Rincón and Neelam 2021). These findings on microbiological quality implications in different farming practices underscore the interconnectedness recognized by the “One-Health concept".

## Conclusions

Overall, the results clearly demonstrate that the endophytic bacterial communities in edible vegetables are diverse, and their structure and functional potential are significantly shaped by the specific vegetable species, the plant organ sampled, and the type of fertilizer management used. The presence of beneficial functional pathways and identified keystone taxa suggests potential avenues for biotechnological applications and contributions to plant health and potentially human well-being. The significant relationship observed between nutrient content, the bacterial community, and predicted function underscores the intricate link between soil management practices, plant health, microbial activity, and the final nutritional quality of vegetables.

While this study provides valuable baseline information, future research should build upon these findings by experimentally validating the predicted functional pathways and the roles of identified keystone taxa. Techniques like metatranscriptomics or metaproteomics could be employed to confirm actual gene expression. Investigating the actual production of toxins, virulence factors, or beneficial compounds by isolated endophytes would provide further clarity and move beyond predictions. Given the potential impact on human health when vegetables are consumed raw, further study is needed to understand the interaction between these vegetable endophytes and the human gut microbiome. This is particularly important concerning the potential transfer of antibiotic resistance genes, which are known to be related to farm practices and have been found in endophytes. Expanding the geographical scope of the study and including a wider range of vegetables and farming systems would also enhance the generalizability of these findings. While the predicted metabolic functions were based on the 16S rRNA gene sequencing profile, further studies are necessary to fully assess the presence and expression of these ecologically important functions of endophytic bacteria.

Despite the associated challenges, differential abundance analysis remains a fundamental tool for understanding microbial community dynamics. Addressing these statistical limitations through improved data handling and analysis methods is vital for advancing ecological research and ensuring the integrity of interpretations drawn from metabarcoding data (Gleason et al 2023; Wirbel et al. 2024).

## Supplementary Information

Below is the link to the electronic supplementary material.

Supplementary file1 (DOCX 79 KB)

Supplementary file2 (JPG 139 KB)

Supplementary file3 (JPG 107 KB)

Supplementary file4 (JPG 61 KB)

Supplementary file5 (JPG 106 KB)

Supplementary file6 (JPG 107 KB)

Supplementary file7 (JPG 218 KB)

Supplementary file8 (JPG 167 KB)

Supplementary file9 (JPG 179 KB)

## Abbreviations

ASV: Amplicon sequence variant
BF: Big farm
CF: Conventional farm
GLM: General linear model
JF: Johannesburg farm
LDA: Linear discriminant analysis
MENA: Molecular ecological network analysis
NMDS: Non-metric multidimensional scaling
NSTI: Nearest Sequenced Taxon Index
OF: Organic farm
PERMANOVA: Permutational multivariate analysis of variance
PERMDISP: Permutational test for homogeneity of multivariate dispersions
PICRUSt2: Phylogenetic investigation of communities by reconstruction of unobserved states 2
QIIME: Quantitative insights into microbial ecology
RDA: Redundancy analysis
RMT: Random matrix theory
SD: Standard deviation
SF: Small farm
SRA: Sequence read archives
TF: Third farm
