# Supplementary material for: High-throughput sequencing analysis of community diversity and functional structure of endophytic bacteria in edible vegetable crops: potential implication on plant microbiological quality
Source: 3 Biotech. 2025 Jun 17;15(7):216. doi: 10.1007/s13205-025-04380-9 (PMC12174041; doi:10.1007/s13205-025-04380-9)
Supplement: Supplementary file 1 — Supplementary file1 (DOCX 79 KB) [file 13205_2025_4380_MOESM1_ESM.docx]

Supplementary information

**High-throughput sequencing analysis of community diversity and functional structure of endophytic bacteria in edible vegetable crops: Potential Implication on plant microbiological quality**

Adekunle Raimi, Rasheed Adeleke^, *^

Unit for Environmental Sciences and Management, North-West University, Potchefstroom, 2520, South Africa

*Corresponding author

Email: [adeleker@arc.agric.za](mailto:adeleker@arc.agric.za), Phone no: +27 012 310 2519

**ORCID**: AR, 0000-0001-6437-6419; RA, 0000-0002-8974-422X.

**Journal:** 3 Biotech Journal

Supplementary Text

Supplementary Text 1

Demultiplexed reads were trimmed of low-quality bases using Trimmomatic software (version 0.38) (Bolger et al. 2014b) by removing poor-quality trailing and leading nucleotide positions from the forward and reverse reads. Afterwards, reads with an average quality score (Phred, Q) below 20 and reads having less than 250 base pair length were expunged. Quality sequence reads were denoised and assembled using DADA2 denoiser (Callahan et al. 2016) in Quantitative Insight into Microbial Ecology (QIIME) software version 2 ((Qiime et al., 2019) to obtain amplicon sequence variants (ASVs). The ASVs taxonomic assignment was performed in QIIME2 software using a pre-trained classifier SILVA rRNA 138 database (Quast et al. 2013; Bokulich et al. 2023). Contaminants (chloroplast, plastids and mitochondria), singletons and non-bacterial sequences such as archaea and those not assigned to any domain were removed from the ASVs count tables before normalization to an even depth, which is a standard of sequence number corresponding to the sample with the least sequences. Multiple sequence alignment was performed to infer the phylogenetic relationship of each of the ASVs and assess the differences of the dominant species among different sample groups.

Supplementary Text 2

The absolute abundance of microbial genera (observed ASVs) and vegetable nutrient content data was uploaded to the molecular ecological network analysis (MENA) pipeline (Deng et al. 2012). Then, the pairwise Pearson correlation was employed to generate correlation coefficients. Using the absolute values of the correlation matrix and the default settings of the pipeline, a similarity matrix with threshold values was generated. Based on the RMT method, an appropriate threshold was applied to the correlation values to convert the similarity matrix into an adjacency matrix, which encodes the strength of the connection between each pair of nodes (Deng et al. 2012). A connection is assumed to have a strong (Pearson’s r > 0.85) and significant (P < 0.01) correlation. Fast greedy modularity optimisation mode was then used to identify submodules within a large module, which were visualised with the Gephi 0.92 (<https://gephi.org>).

Supplementary Text 3

Random networks for each of the phylogenetic molecular ecological networks (pMENs) were constructed based on the Maslov-Sneppen technique, which keeps the numbers of nodes and links constant (Maslov and Sneppen 2002). The random networks and power-law distribution were used to ascertain whether the empirical networks were error-prone and to identify bacterial interactions that were due to non-random patterns, which depict the empirical structure of microbial communities (Pan et al. 2021). The networks were grouped into network hubs, module hubs, connectors (generalists) and peripherals (specialists). The generalists are taxa that are closely connected with others both within and among modules (network hubs), within a module (module hubs) and among different modules within a network (connectors), while the specialists are peripheral taxa with less interaction with other taxa (Guimera and Amaral 2005). The global network properties, individual node centrality, module separation and modularity were evaluated. The modularity of the networks was calculated using MENA and other evaluated topological parameters, including average degree, average path length, clustering coefficient and graph density (Pan et al. 2021).

Supplementary Table 1: Geographical location and climate parameters for the farms

| Farm | Latitude | Longitude | Altitude | Temperature | Precipitation |
| --- | --- | --- | --- | --- | --- |
| BF1 | -26.79578 | 27.03854 | 1,337 | 17.5 | 40.005 |
| BF2 | -26.79668 | 27.03971 | 1,335 | 17.5 | 40.005 |
| BF3 | -26.79724 | 27.03862 | 1,331 | 17.5 | 40.005 |
| BF4 | -26.79821 | 27.03851 | 1,332 | 17.5 | 40.005 |
| BF5 | -26.79468 | 27.03897 | 1,329 | 17.5 | 40.005 |
| SF1 | -26.71509 | 27.08743 | 1,349 | 17.5 | 40.005 |
| SF2 | -26.71299 | 27.08323 | 1,350 | 17.5 | 40.005 |
| SF3 | -26.70109 | 27.08363 | 1,349 | 17.5 | 40.005 |
| SF4 | -26.71624 | 27.09323 | 1,344 | 17.5 | 40.005 |
| SF5 | -26.72739 | 27.08824 | 1,347 | 17.5 | 40.005 |
| JF1 | -26.19619 | 28.06614 | 1,717 | 15.65 | 46.355 |
| JF2 | -26.19419 | 28.07211 | 1,714 | 15.65 | 46.355 |
| JF3 | -26.19619 | 28.07953 | 1,733 | 15.65 | 46.355 |
| JF4 | -26.19475 | 28.06418 | 1,704 | 15.65 | 46.355 |
| JF5 | -26.19497 | 28.06914 | 1,706 | 15.65 | 46.355 |
| TF1 | -26.05436 | 27.67032 | 1,563 | 16.9 | 46.355 |
| TF2 | -26.02465 | 27.79547 | 1,528 | 16.9 | 46.355 |
| TF3 | -26.02458 | 27.69547 | 1,493 | 16.9 | 46.355 |
| TF4 | -26.02465 | 27.59347 | 1,534 | 16.9 | 46.355 |
| TF5 | -26.03465 | 27.67587 | 1,514 | 16.9 | 46.355 |

Altitude (m), precipitation (mm) and temperature (◦C) are annual averages.

Supplementary Table 2: Difference of alpha diversity measure across the fertiliser type factor.

| Alpha diversity | Effect | Test statistic W and sig. P value | r (rank biserial) and 95% CI |
| --- | --- | --- | --- |
| Chao 1 | positive | tiny; W = 2327.00, p = 0.906 | r =0.01, 95% CI [-0.18, 0.20]) |
| Pielou’s evenness | positive | small; W = 2636.00, p = 0.143 | r = 0.15, 95% CI [-0.05, 0.33]) |
| Shannon-Weiner | positive | Small; diff = 0.36, 95% CI [-0.24, 0.97], t (132.96) = 1.19, p = 0.234 | Cohen's d = 0.21, 95% CI [-0.13, 0.54]) |
| Inverse Simpson | positive | small; W = 2618.00, p = 0.166 | r = 0.14, 95% CI [-0.06, 0.32]) |
| Core abundance | positive | very small; W = 2423.00, p = 0.575 | r = 0.05, 95% CI [-0.14, 0.24]) |
| PD | positive | Large; W = 3009.00, p = 0.002 | r = 0.31, 95% CI [0.12, 0.47]) |

Welch's t-test is for testing the group differences mean between the organic fertilised farms and conventional fertilised farms while the Wilcoxon rank sum exact test is for testing the difference in ranks between the OF and CF farms. Effect sizes were labelled following Cohen's (1988) recommendations. Sig. P value; significant P value. PD; phylogenetic diversity, CI; confidence interval. P < 0.05 is statistically significant. Shannon-Weiner was normally distributed and was tested with the Welch t-test.

Supplementary Table 3: Result of Wilcoxon rank sum test with continuity correction testing the difference in ranks between alpha diversity indices and plant organ.

| Alpha diversity | Effect | Test statistic W and sig. P value | r (rank biserial) and 95% CI |
| --- | --- | --- | --- |
| Chao 1 | negative | very large; W = 1382.00, P < 0.001 | r = -0.40, 95% CI [-0.55, -0.22]) |
| Pielou’s evenness | positive | tiny; W = 2400.00, p = 0.678 | r = 0.04, 95% CI [-0.15, 0.23]) |
| Shannon-Weiner | negative | medium; W = 1659.00, p = 0.005 | r = -0.28, 95% CI [-0.45, -0.09]) |
| Inverse Simpson | negative | small (diff = -0.79, 95% CI [-1.38, -0.19], t ((133.64) = -2.63 | Cohen's d = -0.45, 95% CI [-0.79, -0.11]) |
| Core abundance | negative | very small; W = 2167.00, p = 0.534 | r = -0.06, 95% CI [-0.25, 0.13]) |
| PD | positive | very small; W = 2429.00, p = 0.588 | r = 0.05, 95% CI [-0.14, 0.24]) |

Welch's t-test is for testing the group differences mean between the organic fertilised farms and conventional fertilised farms while the Wilcoxon rank sum exact test is for testing the difference in ranks between the OF and CF farms. The effect sizes were labelled following Funder's (2019) recommendations. Sig. P value; significant P value. PD; phylogenetic diversity, CI; confidence interval. p < 0.05 is statistically significant. Shannon-Weiner was normally distributed and was tested with the Welch t-tests.

Supplementary Table 4: Fixed factor interaction effects on the Shannon index

| Variables | Estimate | Std. Error | t value | Pr (>\|t\|) |
| --- | --- | --- | --- | --- |
| (Intercept) | 1.32872 | 0.09119 | 14.571 | < 2e-16 *** |
| Plant lettuce | -0.40107 | 0.14400 | -2.785 | 0.00622 ** |
| Plant onion | 0.04252 | 0.16077 | 0.264 | 0.79185 |
| Plant spinach | -0.29742 | 0.13969 | -2.129 | 0.03528 * |
| Organ root | -0.04876 | 0.13387 | -0.364 | 0.71635 |
| Practice organic | -0.24765 | 0.14607 | -1.695 | 0.09258. |
| Plant lettuce: Organ root | 0.47669 | 0.19810 | 2.406 | 0.01764 * |
| Plant onion: Organ root | 0.30688 | 0.22887 | 1.341 | 0.18251 |
| Plant spinach: Organ root | 0.33947 | 0.19362 | 1.753 | 0.08211. |
| Plant lettuce: Practice organic | 0.19999 | 0.26377 | 0.758 | 0.44981 |
| Plant onion: Practice organic | 0.21646 | 0.21889 | 0.989 | 0.32469 |
| Plant spinach: Practice organic | 0.27378 | 0.20842 | 1.314 | 0.19148 |
| Organ root: Practice organic | 0.06620 | 0.20913 | 0.317 | 0.75213 |
| Plant lettuce: Organ root: Practice organic | -0.79251 | 0.38620 | -2.052 | 0.04234 * |
| Plant onion: Organ root: Practice organic | -0.10202 | 0.31031 | -0.329 | 0.74291 |
| Plant spinach: Organ root: Practice organic | -0.19957 | 0.29500 | -0.676 | 0.50003 |

Degree of freedom - Df, (*) statistical significance code: 0 ‘***’ 0.001 ‘**’ 0.01 ‘*’ 0.05 ‘.’ 0.1 ‘’ 1. The Poisson model (estimated using ML) was fitted to predict Shannon-Weiner diversity with Plant, Organ, and Practice factors. The model's explanatory power is substantial (Nagelkerke's R2 = 0.35) and the model's intercept, corresponding to Plant = cabbage, Organ = leaf and Practice = conventional, is at 1.33 (95% CI [1.14, 1.50], t (120) = 14.57, p < .001).

Supplementary Table 5: PerMANOVA results based on weighted and unweighted Unifrac distances using the bacterial community structure abundance data.

| Fixed factors | Weighted Unifrac (Composition) | | | | Unweighted Unifrac (Composition) | | | |
| --- | --- | --- | --- | --- | --- | --- | --- | --- |
|  | PERMANOVA | | | PERMDISP | PERMANOVA | | | PERMDISP |
|  | Df | R^2^ (%) | P | P | Df | R^2^ (%) | P | P |
| Plant | 3 | 5.65 | 0.002* | 0.069 | 3 | 4.76 | 0.001* | 0.519 |
| Organ | 1 | 1.35 | 0.025* | 0.022 | 1 | 1.11 | 0.005* | 0.634 |
| Fertiliser | 1 | 0.32 | 0.990 | 0.007 | 1 | 0.78 | 0.168 | 0.972 |
| Plant: organ | 3 | 1.75 | 0.800 | 0.021 | 3 | 2.19 | 0.036* | 0.454 |
| Plant: Fertiliser | 3 | 2.34 | 0.321 | 0.063 | 3 | 2.33 | 0.137 | 0.013 |
| Organ: Fertiliser | 1 | 0.55 | 0.694 | 0.002 | 1 | 0.69 | 0.504 | 0.827 |
| Plant: organ: Fertiliser | 3 | 2.06 | 0.512 | 0.048 | 3 | 2.28 | 0.186 | 0.005 |
| Residue | 120 | 85.98 |  |  | 120 | 85.86 |  |  |

Df- degree of freedom, (*) shows statistical significance with P < 0.05, P-value is based on 999 permutations using the adonis () function of the vegan package in R.

Supplementary Table 6: Linear discriminant analysis of significant features between vegetable species

| Features | LDA | P. unadj | P.adj | Significance |  |
| --- | --- | --- | --- | --- | --- |
| Gammaproteobacteria | lettuce | 5.18857 | 0.00092 | 0.02973 | * |
| Pseudomonadaceae | lettuce | 5.09971 | 0.00048 | 0.02012 | * |
| Pseudomonas | lettuce | 5.09971 | 0.00048 | 0.02012 | * |
| Pseudomonadales | lettuce | 5.09205 | 0.00061 | 0.02372 | * |
| Thermoleophilia | onion | 4.49856 | 0.00000 | 0.00090 | *** |
| Enterobacteriaceae | spinach | 4.32607 | 0.00149 | 0.04521 | * |
| Acidimicrobiia | cabbage | 4.22590 | 0.00028 | 0.01420 | * |
| Gaiellales | cabbage | 4.21624 | 0.00000 | 0.00011 | *** |
| Solirubrobacterales | cabbage | 4.21546 | 0.00019 | 0.01064 | * |
| Chloroflexi | onion | 4.12015 | 0.00029 | 0.01443 | * |
| Gaiellales | cabbage | 4.11846 | 0.00000 | 0.00011 | *** |
| Acidobacteriota | onion | 4.09665 | 0.00068 | 0.02527 | * |
| Solirubrobacter | cabbage | 4.05029 | 0.00009 | 0.00755 | ** |
| Solirubrobacteraceae | cabbage | 4.04708 | 0.00140 | 0.04373 | * |
| Vicinamibacteria | cabbage | 4.02487 | 0.00004 | 0.00641 | ** |
| Vicinamibacterales | cabbage | 3.98905 | 0.00024 | 0.01262 | * |
| Beijerinckiaceae | cabbage | 3.95371 | 0.00088 | 0.02944 | * |
| Xanthobacteraceae | cabbage | 3.91966 | 0.00045 | 0.02012 | * |
| Nocardioides | cabbage | 3.90861 | 0.00042 | 0.01980 | * |
| Chloroflexia | cabbage | 3.88187 | 0.00001 | 0.00190 | ** |
| Solirubrobacter | cabbage | 3.87913 | 0.00010 | 0.00793 | ** |
| Microvirga | cabbage | 3.85965 | 0.00001 | 0.00279 | ** |
| Sphingomonas | cabbage | 3.81703 | 0.00008 | 0.00755 | ** |
| Ilumatobacteraceae | cabbage | 3.79741 | 0.00070 | 0.02527 | * |
| Thermomicrobiales | cabbage | 3.78934 | 0.00006 | 0.00641 | ** |
| Sphingomonas | cabbage | 3.77170 | 0.00085 | 0.02944 | * |
| Vicinamibacteraceae | cabbage | 3.76228 | 0.00014 | 0.00981 | ** |
| JG30-KF-CM45 | cabbage | 3.76107 | 0.00006 | 0.00641 | ** |
| Microvirga | cabbage | 3.74349 | 0.00002 | 0.00291 | ** |
| Xanthobacteraceae | cabbage | 3.73937 | 0.00013 | 0.00981 | ** |
| IMCC26256 | cabbage | 3.62420 | 0.00018 | 0.01047 | * |
| Desulfobacterota | cabbage | 3.60551 | 0.00061 | 0.02372 | * |
| Ilumatobacteraceae | cabbage | 3.54840 | 0.00089 | 0.02944 | * |

P.adj; P value adjusted, P. unadj; P value unadjusted * -- 0.05, ** -- 0.01.

**Supplementary Figure legend**

Supplementary Fig. 1: Selected vegetable nutrient content across the vegetable crops and the statistical significance of their mean values represented with either a, b, c or d to show the main effects across the crop species.

Supplementary Fig. 2: Alpha diversity measures across fertiliser types (conventional and organic) and organ type (leaf and root) for A) Chao1, B) Shannon-Weinerer index, C) Pielou’s evenness, and D) Simpson index.

Supplementary Fig. 3: Alpha diversity measure across the plant species showing A) Phylogenetic diversity B) Core abundance

Supplementary Fig. 4: Rarefaction cure plot for amplicon sequence variants (ASVs) of plant species from organic and conventional fertilised farms. The rarefaction was subsampled at a depth of 5000.

**Supplementary Fig. 5:** Relative abundance of endophytic bacteria (> 1% relative abundance) at the a) Dominant phylum and b) Dominant genus taxa. Org; organic fertiliser farm and Conv; conventional fertiliser farm. The relative abundance at the genus level was computed amongst the bacterial communities with above 1% relative abundance after removing the unclassifiable and non-culturable bacterial communities from the data. Each taxon is represented by a different colour while the height represents the relative abundance of the taxon in each sample.

**Supplementary Fig. 6**: Relationship between Shannon-Weiner diversity index and vegetable nutrient content

**Supplementary Fig. 7**: Relationship between Chao 1 index and vegetable nutrient content

**Supplementary Fig. 8**: Relationship between Pielou’s evenness diversity and vegetable nutrient content
