# Supplementary figures and images for: High-throughput sequencing analysis of community diversity and functional structure of endophytic bacteria in edible vegetable crops: potential implication on plant microbiological quality
Source: 3 Biotech. 2025 Jun 17;15(7):216. doi: 10.1007/s13205-025-04380-9 (PMC12174041; doi:10.1007/s13205-025-04380-9)

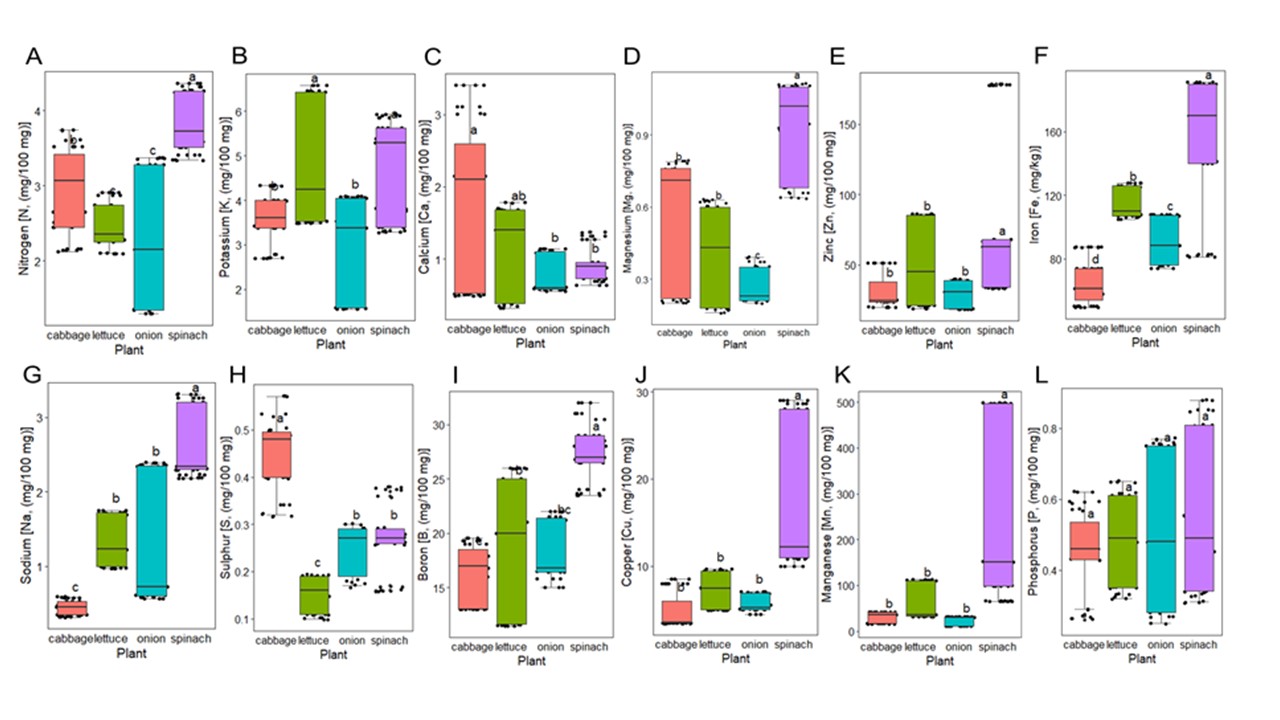

Supplement: Supplementary file 2 — Supplementary file2 (JPG 139 KB) [file 13205_2025_4380_MOESM2_ESM.jpg]

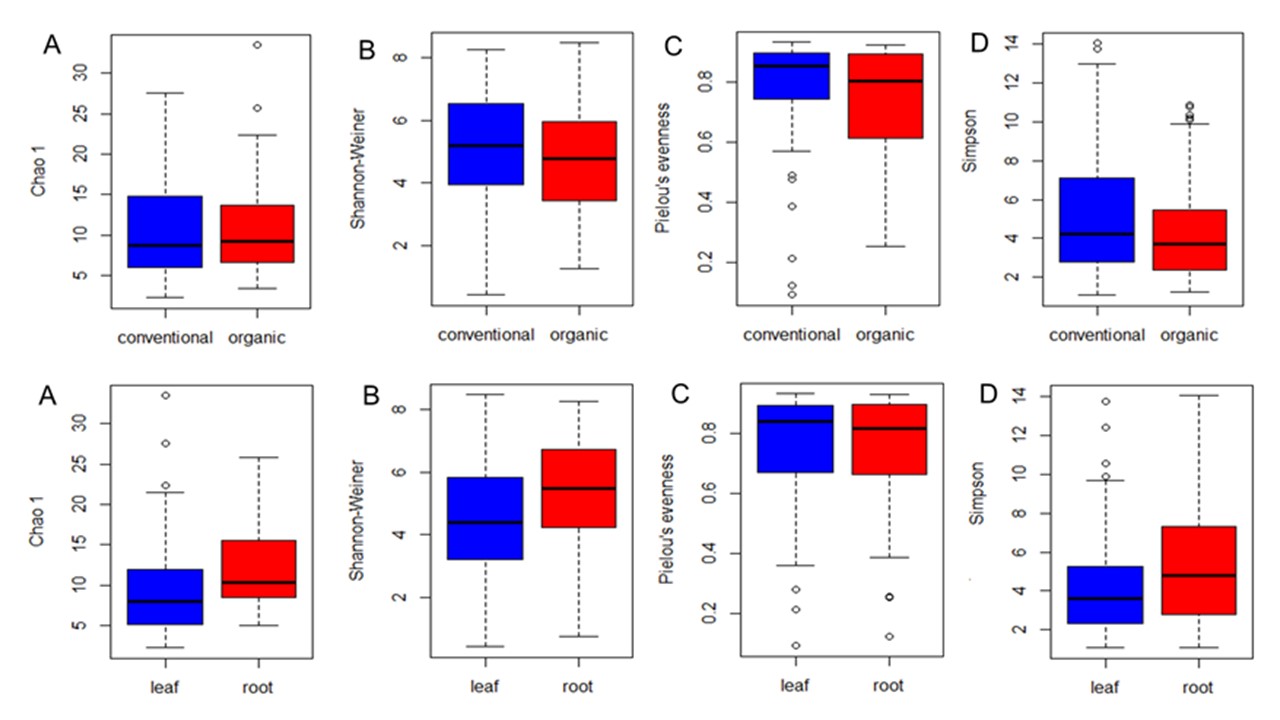

Supplement: Supplementary file 3 — Supplementary file3 (JPG 107 KB) [file 13205_2025_4380_MOESM3_ESM.jpg]

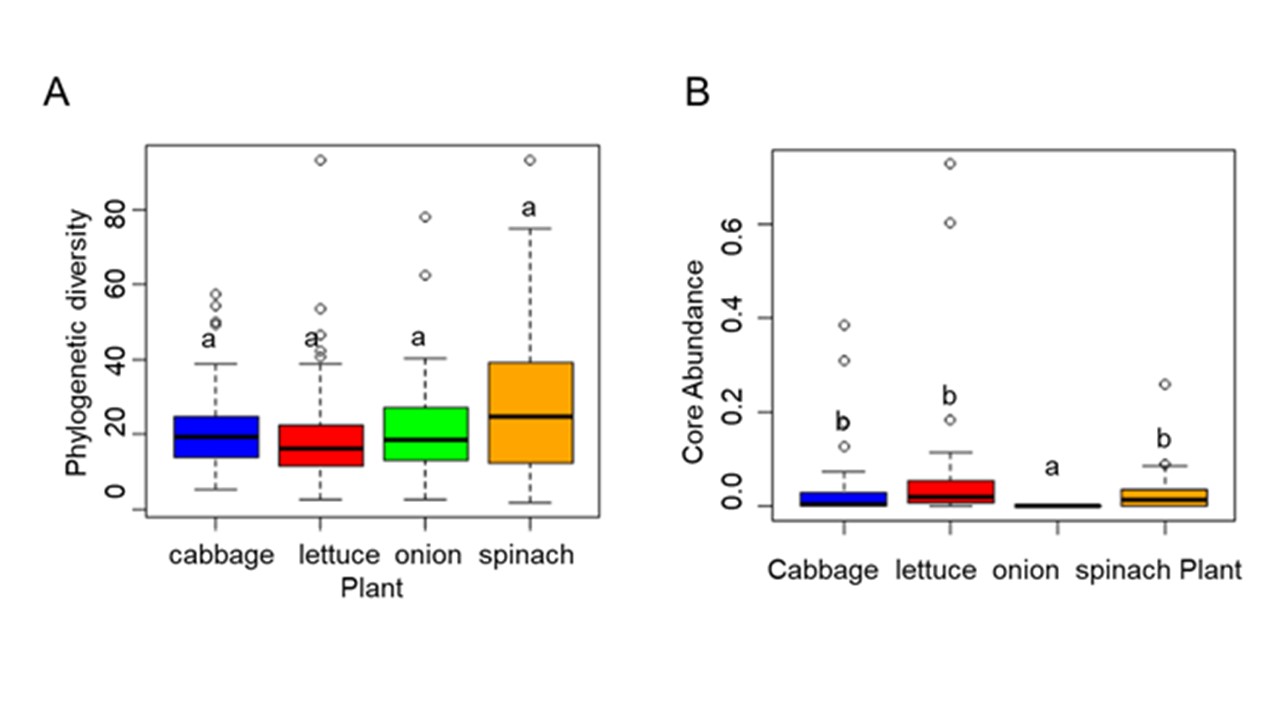

Supplement: Supplementary file 4 — Supplementary file4 (JPG 61 KB) [file 13205_2025_4380_MOESM4_ESM.jpg]

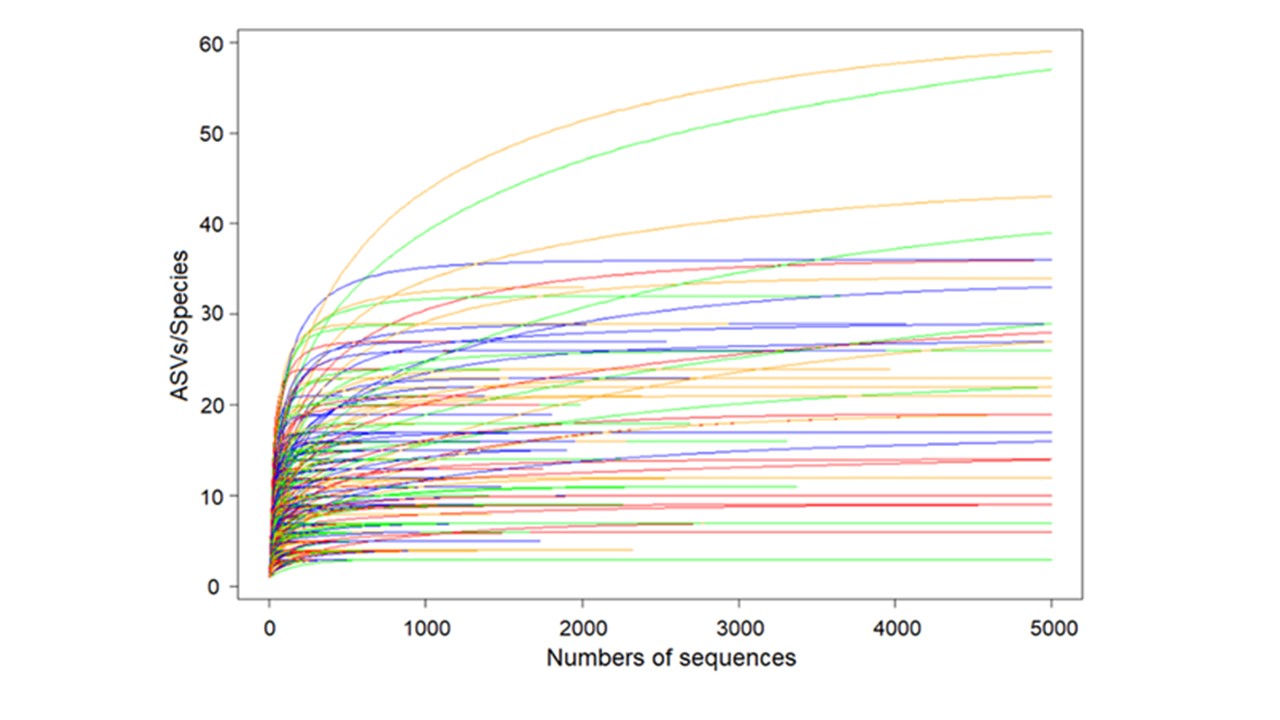

Supplement: Supplementary file 5 — Supplementary file5 (JPG 106 KB) [file 13205_2025_4380_MOESM5_ESM.jpg]

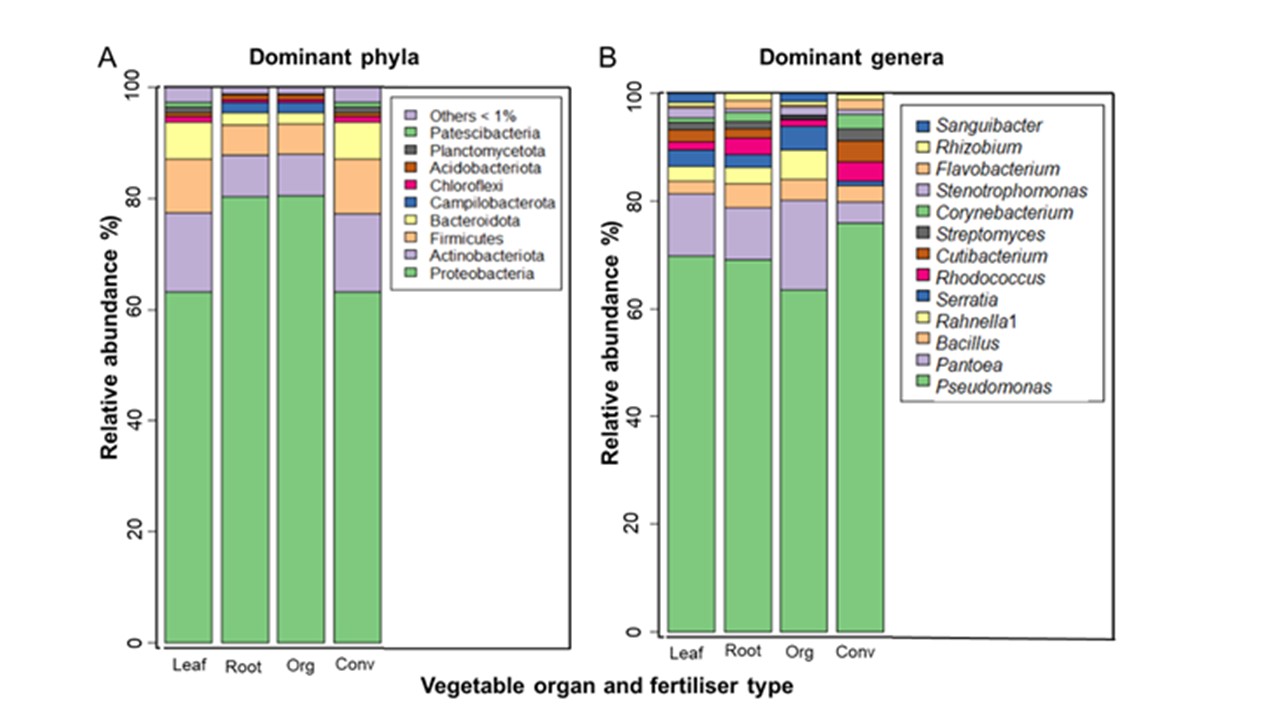

Supplement: Supplementary file 6 — Supplementary file6 (JPG 107 KB) [file 13205_2025_4380_MOESM6_ESM.jpg]

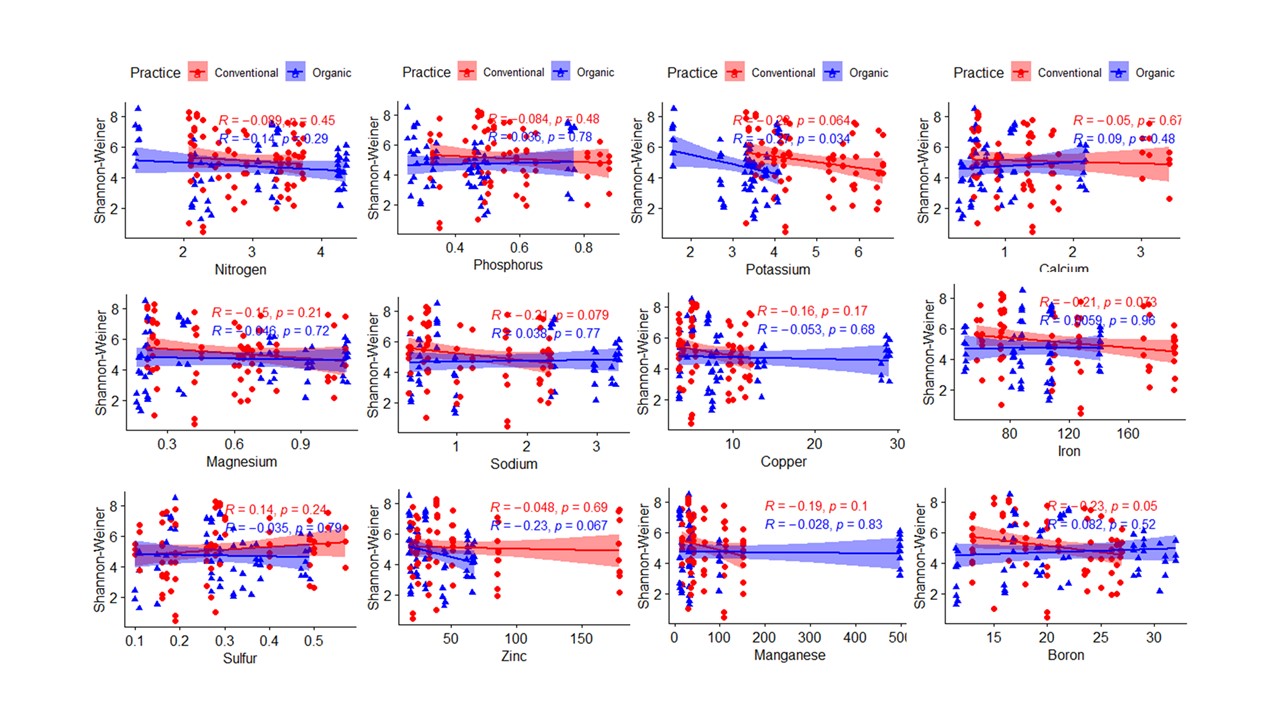

Supplement: Supplementary file 7 — Supplementary file7 (JPG 218 KB) [file 13205_2025_4380_MOESM7_ESM.jpg]

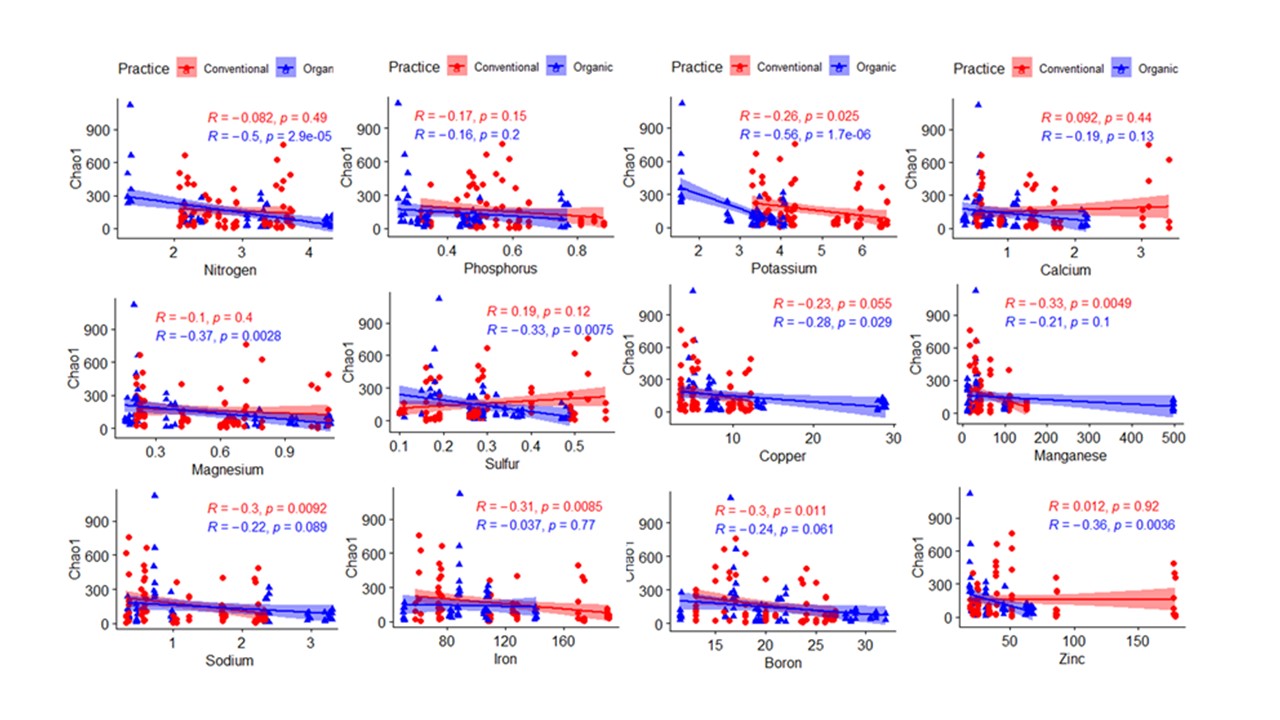

Supplement: Supplementary file 8 — Supplementary file8 (JPG 167 KB) [file 13205_2025_4380_MOESM8_ESM.jpg]

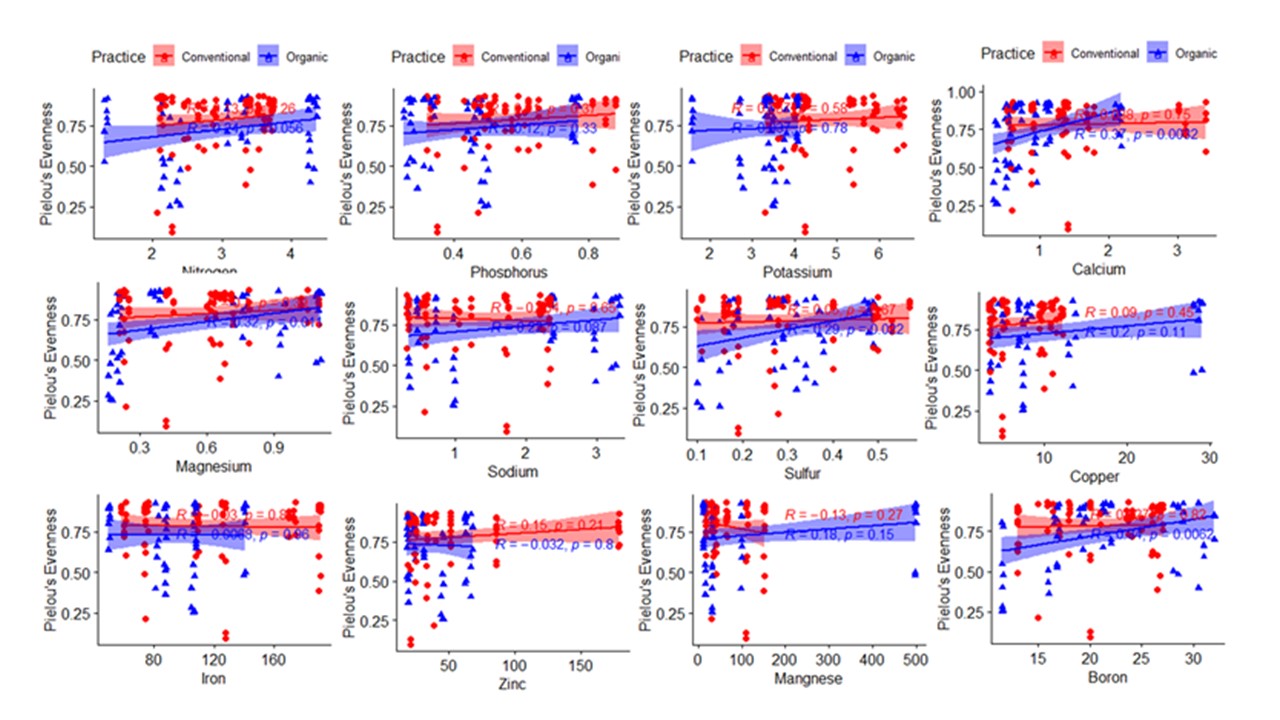

Supplement: Supplementary file 9 — Supplementary file9 (JPG 179 KB) [file 13205_2025_4380_MOESM9_ESM.jpg]
